# From heat-treatment maps to repairable tooling of LPBF-produced M789 maraging stainless steel

**DOI:** 10.1016/j.isci.2026.117196

**Published:** 2026-09-01

**Authors:** Ludmila Kučerová, Michal Ackermann, Andrea Málková, Jiří Šafka, Pavel Žlábek, Michal Kaufman

**Affiliations:** 1 Regional Technological Institute, Faculty of Mechanical Engineering, University of West Bohemia, Univerzitní 8, 301 00 Plzen, Czech Republic; 2 The Institute for Nanomaterials, Advanced Technologies and Innovation, Technical University of Liberec, Studentská 1402/2, 461 17 Liberec, Czech Republic

**Keywords:** additive manufacturing, laser powder bed fusion, M789 steel, heat treatment, long-term stability, additive repairs

## Abstract

Laser powder bed fusion (LPBF) can produce corrosion-resistant, ultra-high-strength maraging stainless steels for tooling, but post-processing routes balancing strength, toughness, thermal stability, and repairability remain unclear. We establish an internally consistent heat-treatment map for cobalt-free M789 across direct aging, solution annealing, and combined routes. As-built material exhibited 1.0 GPa ultimate tensile strength, 320 HV10, and Charpy impact energy up to 135 J. Direct aging at 500°C–520°C for 3 h reached 1.87 GPa and 562–572 HV10, matching the conventional 1000°C/1 h + 500°C/3 h route but reducing toughness to 11 J. Exposure at 350°C for up to 2880 h resulted in modest strengthening, ductility loss, and a chemically stratified oxide scale. LPBF redeposition onto printed M789 produced a metallurgically sound repair, supporting additive repair of thermally and mechanically demanding tooling.

## Highlights

• Direct aging of LPBF M789 reaches 1.87 GPa and 562 to 572 HV10
• Solution annealing improves homogeneity but is not required for peak strength
• Long term exposure at 350°C causes modest strengthening and ductility loss
• LPBF redeposition enables metallurgically sound same alloy repair

## Graphical abstract

**Figure:**
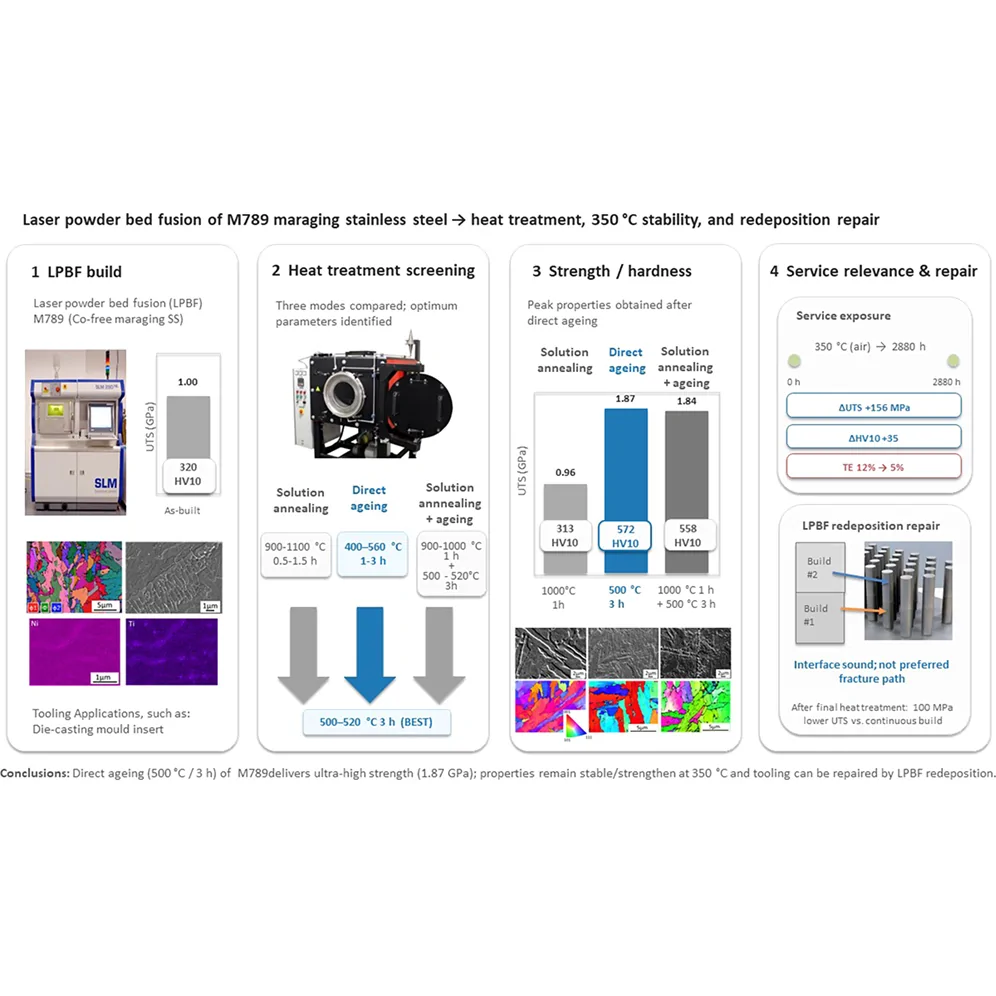

## Teaser

Applied sciences; Materials science

## Introduction

Laser powder bed fusion (LPBF) has moved from a rapid-prototyping tool to a manufacturing route for metallic components in which geometry, local functionality and material efficiency are part of the design. In tooling, the relevant examples include conformally cooled injection-mold inserts, high-pressure die-casting inserts, locally reinforced mold features and repairable high-value tool segments. In these applications, geometric freedom alone is insufficient: the material must retain high strength and hardness under cyclic thermo-mechanical loading, resist corrosion or oxidation in humid cooling channels and chemically active environments, and tolerate local repair or re-deposition.^1^^,^^2^^,^^3^ This application-driven combination of requirements forms the transition from generic additive manufacturing (AM) processability to the specific alloy and post-processing problem addressed here.

Conventional AM tool steels illustrate the compromise. The established benchmark, 18Ni-300 maraging steel (1.2709), can achieve very high strength after aging and is therefore widely used for LPBF tooling; however, its lack of chromium makes it vulnerable to corrosion in water-cooled or chemically aggressive service environments.^4^^,^^5^ Conversely, precipitation-hardening stainless steels such as 17-4 PH provide passivation, but their hardness and strength are generally insufficient for severe cyclic loading in highly stressed dies and molds.^1^^,^^3^ A conformally cooled mold insert or die-casting insert therefore requires a property combination that is difficult to obtain from either alloy family alone: maraging-steel strength, stainless-steel corrosion resistance, adequate toughness, thermal stability and repair compatibility.

M789 maraging stainless steel was developed to close this gap by combining the precipitation-strengthening concept of maraging steels with a chromium-rich stainless matrix.^3^^,^^6^ A defining feature of M789 is its cobalt-free design. In conventional maraging steels, cobalt contributes to precipitation kinetics and strengthening, but its elimination reduces dependence on a costly and safety-sensitive alloying element.^4^^,^^7^ M789 instead relies on titanium and aluminum as the main hardening elements in a low-carbon (<0.02 wt. %), Cr-rich (approximately 12 wt. %) and Ni-rich (approximately 10 wt. %) martensitic matrix.^4^^,^^8^ The ultra-low carbon level is beneficial for LPBF because it reduces susceptibility to cold cracking and delamination during rapid solidification, while still allowing a martensitic matrix to form.^3^^,^^9^

LPBF processing of M789 adds a second level of complexity. Rapid solidification, with cooling rates often reported on the order of 10^6^°C s^−1^, produces a refined cellular or cellular-dendritic substructure, segregation of alloying elements, and a supersaturated martensitic matrix in the as-built (AB) state.^10^^,^^11^ Published examples of LPBF M789 show that the AB material is relatively soft and machinable, but also contains retained austenite, Ti/Ni segregation and Ti- and Al-rich oxide particles inherited from powder processing and repeated laser melting.^2^^,^^3^^,^^8^^,^^12^ These features are not only microstructural descriptors: pores, oxides and surface discontinuities have been linked to reduced ductility, localized corrosion and fatigue initiation in additively manufactured M789 and related maraging steels.^3^^,^^5^^,^^13^ Thus, the post-processing strategy must be assessed through a processing-structure-property-service chain rather than solely by hardness.

The required properties are obtained mainly through heat treatment. According to previous TEM/APT-based and thermodynamic studies on M789 and related Cr-Ni-Ti-Al maraging stainless steels, aging is associated with nanoscale Ni-Ti-Al-rich precipitates, commonly described as η-Ni_3_Ti/Ni_3_(Ti, Al)-type and, in some cases, β-NiAl-type phases. In the present work, these precipitates are not directly phase-identified; their role is inferred from the heat-treatment response and compared with the literature, with aging near 500°C commonly yielding the highest strength and hardness.^4^^,^^14^^,^^15^ The manufacturer-recommended route combines solution annealing at 1000°C with subsequent aging at 500°C, but literature examples also show that direct aging can produce high strength without prior homogenisation.^4^^,^^14^ At the same time, the optimum is narrow: insufficient aging leaves the alloy under-strengthened, whereas excessive temperature or time promotes precipitate coarsening and austenite reversion, reducing the strengthening effect.^4^^,^^16^ Existing studies therefore identify the basic strengthening mechanism, but they do not yet provide a broad, internally consistent tensile and toughness map covering direct aging, solution annealing and combined treatments under identical printing and testing conditions.

For tooling applications, the transition from heat-treatment optimization to service performance is equally important. M789 benefits from its chromium content and forms a passive film, providing substantially better corrosion resistance than 18Ni-300 and behavior closer to that of precipitation-hardening stainless steels.^5^^,^^17^ However, real molds and dies also experience prolonged exposure to elevated temperatures, oxidizing atmospheres, repeated thermal gradients and local wear or damage. Repairability is therefore a practical requirement for high-value tools. Examples of hybrid manufacturing, such as the deposition of M789 onto wrought N709 steel, demonstrate that metallurgical bonding is feasible.^14^ However, they do not directly address whether previously printed M789 can be repaired by subsequent LPBF redeposition of the same alloy, nor whether the repaired interface remains mechanically sound after aging.

These application examples illustrate why the research objective cannot be reduced to a single peak-strength heat treatment. A conformally cooled injection-mold insert must combine resistance to corrosion in cooling channels with dimensional stability, machinability before final aging and sufficient toughness to avoid brittle damage during service. A high-pressure die-casting insert is exposed to repeated thermal gradients, contact with molten non-ferrous alloys and local surface degradation; for such a component, a post-processing route must therefore preserve strength and hardness during prolonged exposure near service temperature, while avoiding excessive loss of ductility. For repairable tool segments, an additional requirement is imposed: the material deposited during a later LPBF step must bond metallurgically to the previously printed material and must not create a weak interface after subsequent aging.

Several observations in the literature support the feasibility of such a framework, but these observations remain fragmented. LPBF-fabricated M789 has already been shown to achieve high hardness after aging, exhibit improved corrosion behavior compared with 18Ni-300, and form sound bonds in dissimilar hybrid builds.^4^^,^^5^^,^^14^^,^^17^ However, these examples address separate parts of the design problem. They do not show, within a single consistent experimental dataset, how to select between direct aging and solution-based routes when strength, toughness, thermal exposure, and same-material repairability must be considered together.

Against this background, the remaining knowledge gap is not whether M789 can be LPBF-processed or precipitation-hardened, but rather how its post-processing should be selected for realistic tooling scenarios. Three linked questions are particularly important. First, systematic datasets on tensile strength, toughness, and hardness are needed to resolve the strength-ductility-toughness trade-off across a broad heat-treatment space, because many available studies focus mainly on hardness or microstructural descriptors. Second, direct comparison of published data is hindered by differences in printing parameters, specimen geometry, gauge length, and surface condition, which are especially critical for elongation and toughness. Third, the response of aged M789 to long-term exposure at service-relevant temperature and the feasibility of same-material LPBF repair have not yet been integrated with heat-treatment optimization. To address these limitations, the present work establishes an extended, internally consistent heat-treatment window encompassing direct aging, solution annealing, and combined routes. It further evaluates long-term thermal stability at 350°C and examines repair by LPBF redeposition onto previously printed M789. By linking post-processing optimization, thermal-exposure response, and repairability, this study provides property-based design guidelines for durable, repairable LPBF tooling operating under demanding thermal and mechanical service conditions.

## Results

### Powder feedstock and experimental workflow

The powder was used as a commercial M789 AMPO feedstock supplied by Böhler Edelstahl. The alloy is a low-carbon, cobalt-free maraging stainless steel containing approximately 12 wt. % Cr, 10 wt. % Ni, 1 wt. % Ti and 0.6 wt. % Al (Table 1). The powder composition is reported as supplier-declared values, whereas the chemical composition, measured by optical emission spark spectrometry on the consolidated LPBF material, is included to document the material actually used in the experimental program. The supplier certificate reports only the nominal contents of the principal alloying elements; residual elements not provided by the supplier are therefore marked as n.r. As the present work focuses on the post-processing response, long-term thermal stability, and LPBF repairability of the consolidated material rather than on powder-lot qualification, no additional chemical analysis of the feedstock was performed.

**Table 1:** Chemical composition in weight% (n.r.—not reported in the supplier certificate; bal.—balance)

	C	Cr	Mo	Ni	Ti	Al
Powder (declared by supplier)	<0.02	12.20	1.00	10.00	1.00	0.60
Measured on the printed parts	0.009	12.04	0.61	10.05	1.10	0.58

	Si	Mn	P	S	V	Fe

Powder (declared by supplier)	n.r.	n.r.	n.r.	n.r.	n.r.	Bal.
Measured on the printed parts	0.44	0.05	0.0066	0.0023	0.026	Bal.

**Figure 1:**
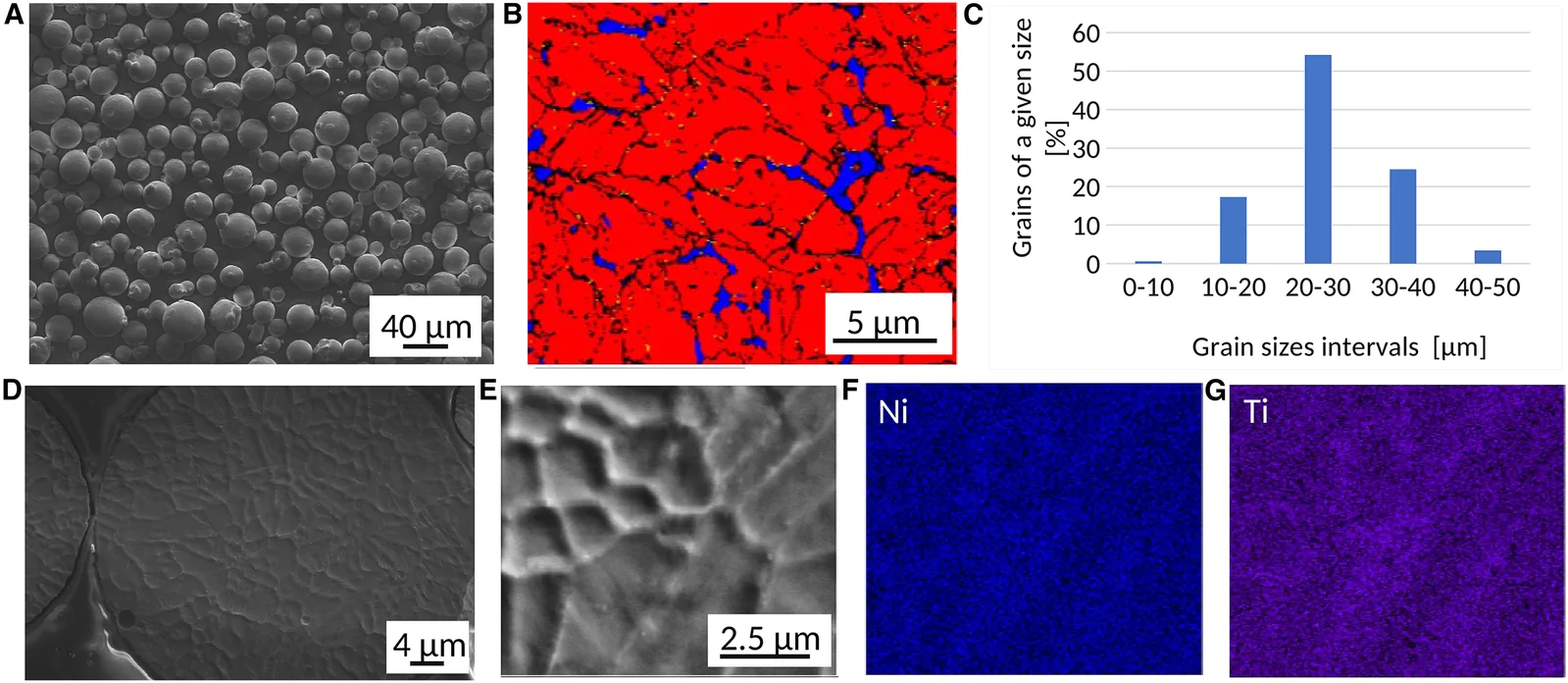
The M789 feedstock is predominantly spherical and exhibits a narrow particle-size distribution with local retained austenite and Ni/Ti segregation (A) Morphology of powder particles. (B) EBSD phase map with retained austenite shown in blue. (C) Histogram of powder particle-size distribution. (D) Cross-section of powder particles. (E) Detail of the cellular solidification structure, and corresponding EDS elemental maps of (F) Ni and (G) Ti obtained from the same region as shown in (E). Scale bars, 40 μm (A), 5 μm (B), 4 μm (D), and 2.5 μm (E–G).

The powder was produced by gas atomization (Figures 1A–1G). Image-based particle-size analysis of individual particles showed a median particle diameter (D50) of 25.3 μm, with D10 = 18.8 μm and D90 = 35.8 μm. The particle-size distribution was therefore relatively narrow, centered at approximately 25–26 μm, consistent with the powder morphology required for stable powder spreading and LPBF processing. The representation of different particle sizes corresponded to a Gaussian distribution (Figure 1C). The particles were predominantly spherical, with a small number of so-called satellites. The powder cross-sections (Figures 1D and 1E) show a cellular structure formed during rapid cooling during atomization. EBSD analysis showed that retained austenite (fcc iron) occurs primarily along the boundaries of the cellular structure (Figure 1B), where it is stabilized by the increased nickel content (Figures 1F and 1G).

**Figure 2:**
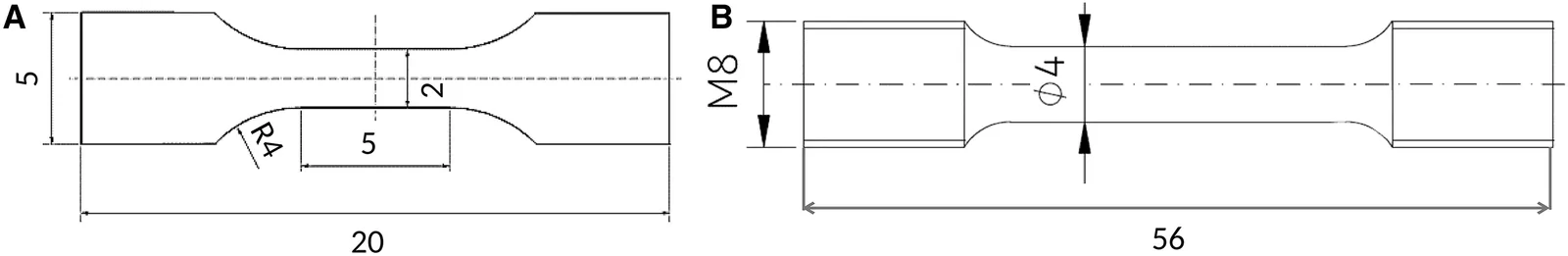
Two tensile-specimen geometries were used to separate heat-treatment screening from repair-interface testing (A) Small flat specimens used for heat-treatment and thermal-stability tests. (B) Larger round specimens with a circular cross-section used for testing hybrid/redeposited samples.

Two tensile-sample geometries were used in the experimental program (Figure 2). Small flat specimens were used for the heat-treatment map and the 350°C stability series. In contrast, larger round specimens were used for the repair/redeposition comparison, ensuring the interface was located within the gauge section without occupying most of the tested volume.

**Figure 3:**
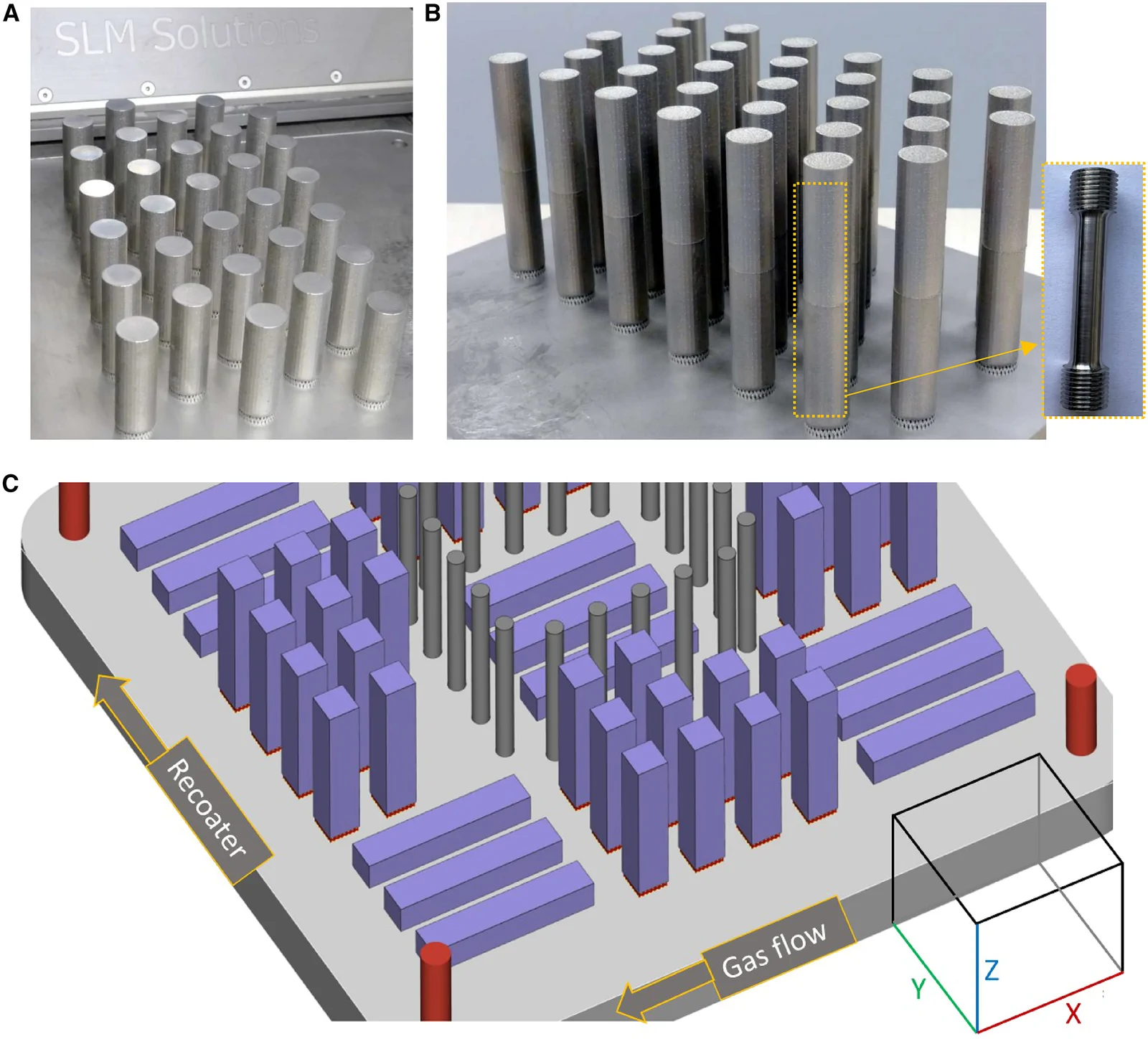
A two-stage LPBF workflow produced hybrid M789 specimens for evaluating same-material repair by redeposition (A) Cylindrical foundations after the first build step. (B) Completed hybrid specimens after redeposition, including a machined tensile specimen. (C) Specimen orientation and placement on the build platform.

The LPBF repair/redeposition workflow is summarized in Figure 3. A first M789 build was removed from the machine, ground, cleaned, reinserted and aligned before redeposition of the same alloy, producing hybrid samples used to assess the feasibility of same-material additive repair.

### As-built material

**Figure 4:**
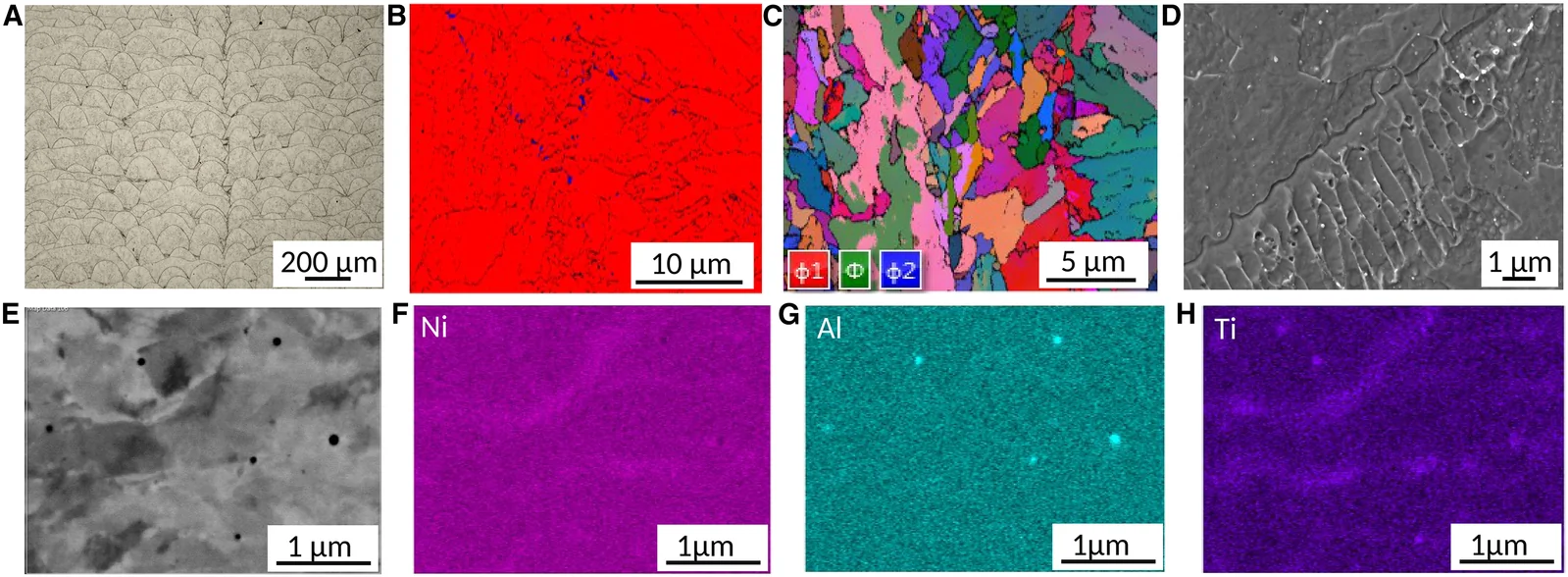
As-built LPBF M789 has a fine, predominantly martensitic microstructure with local retained austenite, cellular segregation, and Ti/Al-rich oxide inclusions (A) Representative light micrograph shows melt pools. (B) Representative EBSD phase map shows retained austenite in blue and the martensitic matrix in red. (C) Representative EBSD Euler map with grain boundaries in black. (D) Representative SEM detail of the cellular microstructure near a melt-pool boundary. (E) BSE image of the area selected for EDS analysis and corresponding EDS maps of (F) Ni, (G) Al, and (H) Ti. (A–D) show representative but non-identical regions; (E–H) were acquired from the same region. The Al-bearing feature in (G) is a Ti/Al-rich oxide inclusion. Scale bars, 200 μm (A), 10 μm (B), 5 μm (C), and 1 μm (D–H).

The microstructure exhibited several features typical of additively manufactured materials. These included traces of the laser scan strategy, visible as half-elliptical melt pools in Figure 4A, and segregation of alloying elements along grain boundaries (Figures 4E, 4F, and 4H). In addition, small amounts of aluminium- and titanium-rich oxides were detected, as confirmed by EDS analysis (Figures 4E–4G and 4H). Notably, the cellular microstructure commonly observed throughout the volume of AM steels was only locally present in M789. It was confined to limited regions, mainly near melt pool boundaries (Figure 4D).

The microstructure is predominantly martensitic (red), with only a small fraction of retained austenite (blue) (Figure 4B). Compared with the powder feedstock, the amount of retained austenite is markedly reduced after AM. Many grains exhibit elongated morphology, likely due to thermal gradients during solidification. The AB material also shows a relatively fine grain size (Figure 4C), consistent with the rapid solidification rates inherent to the SLM process. The mean linear intercept length for the grains evaluated in two perpendicular directions was 1.64 μm and 1.58 μm, indicating no pronounced directional difference. The corresponding standard deviations of the individual intercept lengths were 1.55 μm and 1.50 μm, respectively, reflecting the broad grain-size distribution of the LPBF microstructure rather than uncertainty in the mean value.

Mechanical properties were determined in the three principal build directions to evaluate material isotropy (Table 2). Because two tensile-sample geometries were used, the AB reference properties were established for both. In the AB condition, the larger round samples exhibited yield tensile strengths of 753–793 MPa, ultimate tensile strengths of 1015–1019 MPa, and total elongations of 15–16%, depending on build direction. The small flat samples showed lower yield and ultimate tensile strengths of 691–719 MPa and 982–988 MPa, respectively, but a markedly higher elongation of 23–24%. The mechanistic origin of this geometry-induced offset is discussed in the mechanical properties section; in brief, the higher stress triaxiality in the round geometry promotes earlier damage onset and reduces measured elongation, while small-flat samples are additionally more sensitive to surface and alignment effects. Overall, both sample sets indicated a high degree of isotropy in the printed part. A one-way ANOVA across the X, Y, and Z directions on the small-flat dataset yielded F(2, 12) ≤ 2.1 (*p* ≥ 0.17) for yield strength, ultimate tensile strength, and elongation, supporting the conclusion of build-direction isotropy. In the AB condition, M789 exhibited relatively high and fairly isotropic impact toughness. The absorbed energy increased slightly from 116 ± 19 J in the X direction to 132 ± 21 J and 135 ± 14 J in the Y and Z directions, respectively. Given the overlap in standard deviations, the differences between orientations appear modest, indicating limited anisotropy in impact behavior after printing. Hardness was approximately 320 HV10. The yield-strength standard deviation of the Z-direction larger round samples used as the continuous reference in Tables 4, 5, and 6 was 115 MPa, while the X and Y directions showed similarly wide scatter of 108 MPa (Table 2). The scatter is mainly limited to the 0.2% proof/yield strength; the same Z-direction round specimens showed a much narrower variation in UTS (1015 ± 9 MPa) and total elongation (15 ± 1%). This indicates that the variability is associated primarily with the onset of plastic deformation rather than with final tensile failure. Several factors may contribute, including residual-stress gradients, local microstructural heterogeneity and the statistical distribution of pores or Ti/Al-rich oxide inclusions within the larger gauge volume.

**Table 2:** Mechanical properties of the as-built sample

Small flat samples				Larger samples with circular cross-section			
	YTS [MPa]	UTS [MPa]	TE [%]		YTS [MPa]	UTS [MPa]	TE [%]
X	713 ± 20	986 ± 5	24 ± 1	X	793 ± 108	1019 ± 9	15 ± 1
Y	691 ± 26	988 ± 6	23 ± 1	Y	756 ± 108	1015 ± 13	16 ± 1
Z	719 ± 22	982 ± 3	24 ± 1	Z	753 ± 115	1015 ± 9	15 ± 1

	Impact toughness [J]				Hardness HV 10 [-]		

X	116 ± 19			X	320 ± 3		
Y	132 ± 21			Y	319 ± 3		
Z	135 ± 14			Z	323 ± 3

**Table 4:** Comparison of the properties of the continuously printed part and the hybrid part, both without heat treatment and measured using larger samples with a circular cross-section

	YTS [MPa]	UTS[MPa]	TE [%]
Continuous reference 3D print (without HT)	753 ± 115	1015 ± 9	15 ± 1
Hybrid part (without HT)	766 ± 6	1018 ± 3	14 ± 1

For comparison, the properties in the Z (build) direction were also evaluated using larger samples.

**Table 5:** Mechanical properties of hybrid parts after heat-treatment and reference properties of a part printed at the same time with the same heat-treatment

	YTS [MPa]	UTS [MPa]	TE [%]	HV10 [-]
Reference continuously printed part, 500°C/3 h	1789 ± 24	1872 ± 21	4 ± 1	562 ± 4
Hybrid part, 500°C/3 h	1672 ± 30	1771 ± 20	4 ± 2	570 ± 21
Reference continuously printed part 1000°C/1 h, 500°C/3 h	1742 ± 30	1839 ± 54	3 ± 2	558 ± 7
Hybrid part, 1000°C/1 h, 500°C/3 h	1575 ± 23	1729 ± 9	5 ± 1	554 ± 15

All values were obtained from larger round specimens with circular cross-section; hybrid samples are compared only with continuously printed references of the same geometry.

**Table 6:** Comparison of the mechanical properties of M789 and 18Ni-300 (only larger round specimens with circular cross-section are used for literature/datasheet benchmarking to avoid mixing tensile geometries)

Heat-treatment (if any)	YTS [MPa]	UTS [MPa]	TE [%]	HV10 [–]
As-built (Table 2)	753 ± 115	1015 ± 9	15 ± 1	321 ± 2
500°C/3 h (Table 5)	1789 ± 24	1872 ± 21	4 ± 1	562 ± 4
1000°C/1 h + 500°C/3 h (Table 5)	1742 ± 30	1839 ± 54	3 ± 2	558 ± 7
1000°C/1 h + 500°C/3 h^6^	1670–1770	1800–1900	4–8	531–567^a^
1000°C/1 h + 500°C/3 h^3^	1607 ± 26	1610 ± 28	2 ± 0.1	549^a^
18Ni-300 (480°C/5 h)^9^	1998 ± 32	2217 ± 73	2 ± 0.3	653

a Recalculated from HRC values.

### Heat-treated samples

**Figure 5:**
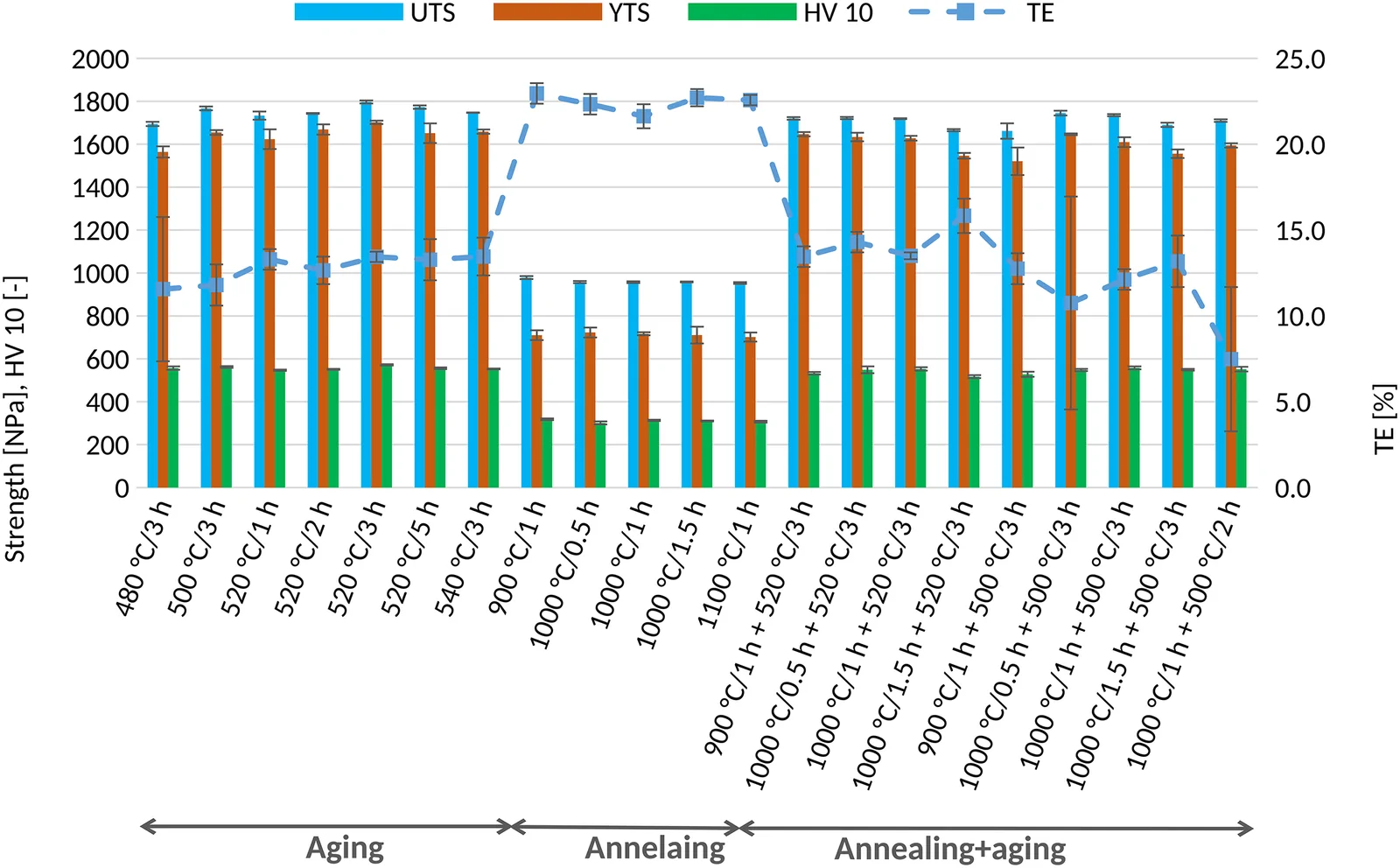
Direct aging at 500°C–520°C for 3 h provides the highest strength and hardness within the heat-treatment map Mechanical properties were measured exclusively on small flat specimens and should be compared only within this geometry. UTS, YTS, and HV10 are plotted against the left y axis; total elongation (TE) is plotted against the right y axis. Data are mean ± SD (*n* = 5 specimens per condition); in some cases, error bars are smaller than the symbols or bar widths.

**Figure 6:**
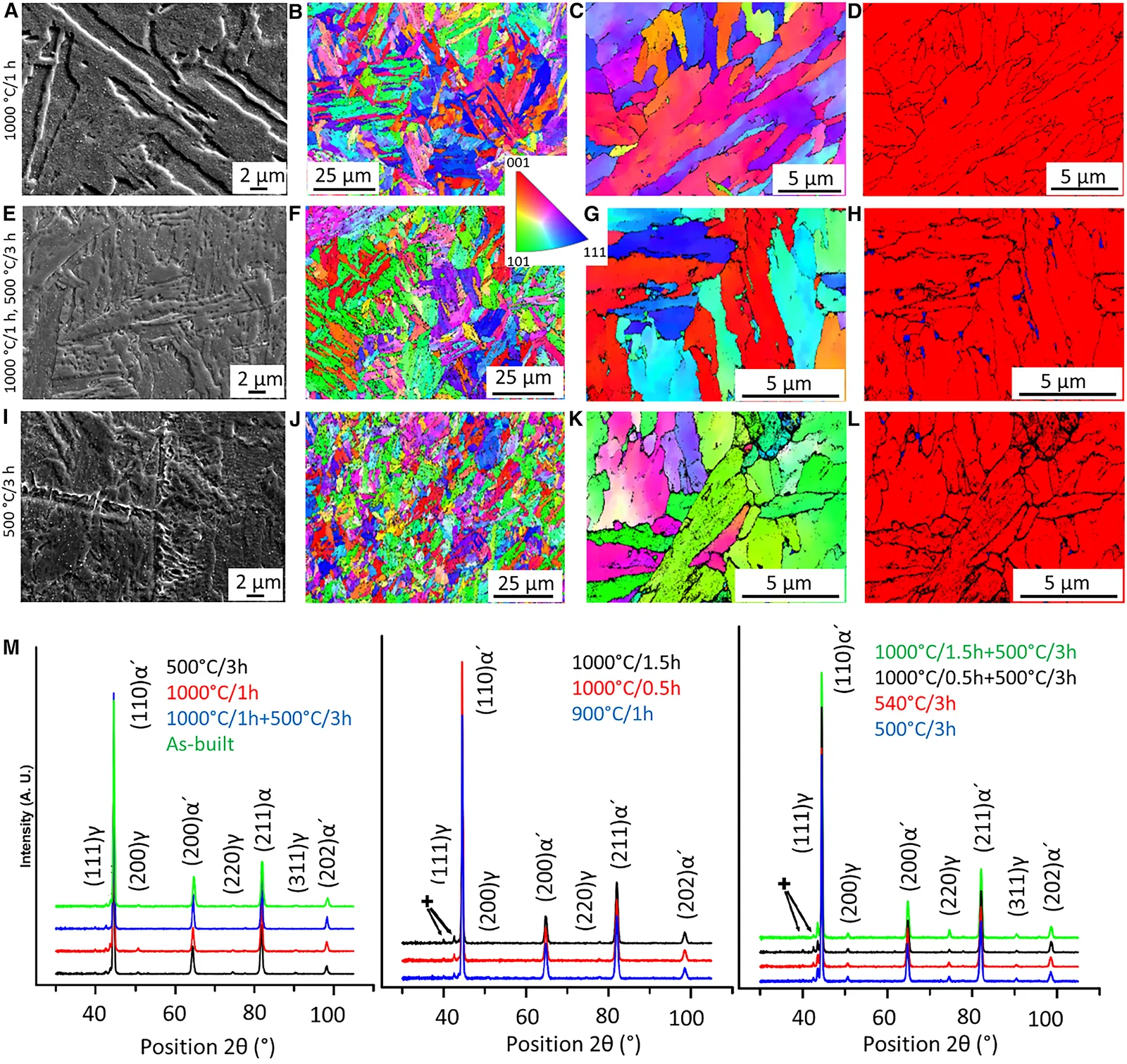
Solution annealing homogenizes the LPBF microstructure, whereas subsequent or direct aging produces a strengthened martensitic state containing retained austenite (A–D) Solution annealing at 1000°C for 1 h. (E–H) Solution annealing at 1000°C for 1 h followed by aging at 500°C for 3 h. (I–L) Direct aging at 500°C for 3 h. Within each row, panels show an SEM image, an EBSD inverse-pole-figure (IPF) map at lower magnification, an IPF map at higher magnification, and an EBSD phase map with martensite in red and retained austenite in blue. (M) XRD patterns of selected as-built and heat-treated states. Scale bars, 2 μm (A, E, I), 25 μm (B, F, J), and 5 μm (C, D, G, H, K, L).

In the case of solution annealing alone, the material’s strength and hardness are significantly lower (see Figure 5), as no precipitation strengthening occurs. The complete numerical dataset underlying Figure 5 is provided in Table S1. Instead, the process leads to homogenization of the structure and the removal of AM marks, making the steel more similar to conventionally processed steel, as shown in Figures 6A–6D. Within the solution-annealing-only subset, the explored changes in annealing temperature and holding time did not produce practically relevant strengthening. Yield strength was statistically unchanged, F(4, 20) = 0.51, *p* = 0.731. Although small statistical differences were detected in ultimate tensile strength, elongation, and hardness, their absolute ranges remained narrow: 953–978 MPa for ultimate tensile strength, 21.6–23.0% for elongation, and 301–318 HV10. The solution-annealing step, therefore, mainly homogenized the LPBF microstructure and removed AM-related features (Figures 6A–6D), rather than providing precipitation strengthening. The degree of homogenization of the structure was not affected, as homogenization and the removal of AM marks occurred even at 900°C. Higher ductility is associated with the homogenization of the structure and the elimination of the printed substructure.^18^^,^^19^^,^^20^^,^^21^ In the case of the 900°C/1 h regime, less than 2% of retained austenite was measured in the resulting microstructure (Figure 6M). For clarity, the complete XRD-derived phase fractions are provided in Table S2. Because no additional crystalline phase was reliably quantifiable from the diffraction patterns, the remaining fraction was assigned to the α/α′ Fe-rich martensitic matrix. Throughout this work, the retained-austenite fraction was determined by X-ray diffraction using full-pattern Rietveld refinement in TOPAS 5, as described in the STAR Methods. EBSD phase maps were used as a complementary qualitative check of the spatial distribution. After annealing at 1000°C or higher, no measurable proportion of tained austenite remained in the microstructure, as shown for the 1000°C/1 h solution-annealed state in the corresponding EBSD phase map (Figure 6D).

Among the direct-aging treatments, the highest strength was achieved after aging at 520°C for 3 h, reaching a tensile strength of 1797 MPa, a hardness of 572 HV10, and an elongation of 13%, as shown in Figure 5. In terms of holding time, shorter durations did not provide sufficient strengthening, whereas longer holds caused a gradual reduction in strength. A similar dependence was observed for aging temperature: lower temperatures resulted in less pronounced strengthening, while higher temperatures promoted over-aging and a further decrease in strength. Reducing the aging temperature to 500°C resulted in only a minor decrease in tensile strength to 1767 MPa and hardness to 562 HV10.

To support the interpretation of Figure 5, a one-way ANOVA was performed on the heat-treatment dataset obtained from small flat specimens. Across all heat-treatment regimes, the treatment condition had a highly significant effect on all measured properties: ultimate tensile strength, F(23,96) = 4495.2, *p* < 0.001; yield strength, F(23,96) = 1130.4, *p* < 0.001; total elongation, F(23,96) = 23.3, *p* < 0.001; and hardness, F(23,96) = 1195.2, *p* < 0.001. When the heat-treatment families were analyzed separately, direct aging showed the strongest effect on the mechanical response, with F(9,40) = 1774.0, 268.3, 12.8, and 746.8 for ultimate tensile strength, yield strength, elongation, and hardness, respectively (*p* < 0.001 for all). This confirms that precipitation hardening during aging is the dominant factor controlling the strength and hardness of LPBF-produced M789.

Microstructurally, the laser-scan traces remained apparent after aging, with melt-pool boundaries still visible (Figure 6I), similar to the AB condition. At higher magnification, electron microscopy revealed a cellular substructure characteristic of AM (Figures 6I–6L). Although not resolved in the present micrographs of the etched microstructure, strengthening during aging is attributed to the formation of nanoscale precipitates that impede dislocation motion. As reported in the literature,^2^^,^^4^^,^^8^ these precipitates are commonly Ni-Ti-Al-rich particles, often described as η-Ni_3_(Ti, Al).

The two-step heat treatment combined solution annealing and subsequent aging to promote microstructural homogenization, followed by a precipitation-strengthened state (Figures 6E–6H). The nanoscale precipitates responsible for this strengthening are not directly resolved in Figure 6; their presence is inferred from the mechanical response and from the established aging behavior of M789 reported in the literature. The resulting mechanical properties were comparable to those obtained after aging alone. The highest ultimate tensile strength and yield strength were achieved for 1000°C/1 h + 500°C/3 h and, similarly, for 1000°C/0.5 h + 500°C/3 h. This indicates that the solution-annealing step can likely be reduced by half without compromising strength, potentially offering economic benefits. For all heat-treatment conditions (including direct aging), the martensitic matrix contained approximately 5–7% retained austenite. Direct aging and the two-step route with aging at 500°C produced comparable retained-austenite levels (5.8% and 5.1–6.6%, respectively; Figure 6M; Table S2).

The combined solution-annealing plus aging routes also showed statistically significant differences among individual schedules, F(8,36) = 22.8 for ultimate tensile strength, 16.6 for yield strength, 4.10 for elongation, and 11.1 for hardness (*p* ≤ 0.0015). However, their property window remained close to the peak under direct aging conditions, supporting the conclusion that prior solution annealing is not essential when the primary objective is maximum strength and hardness.

After solution annealing at 1000°C for 1 h, the measured notch toughness was 133 ± 13 J, which is comparable to the AB value of 135 ± 14 J in the Z direction. Subsequent aging in a two-step regime, however, led to a pronounced reduction in toughness to 8 ± 1 J.

**Figure 7:**
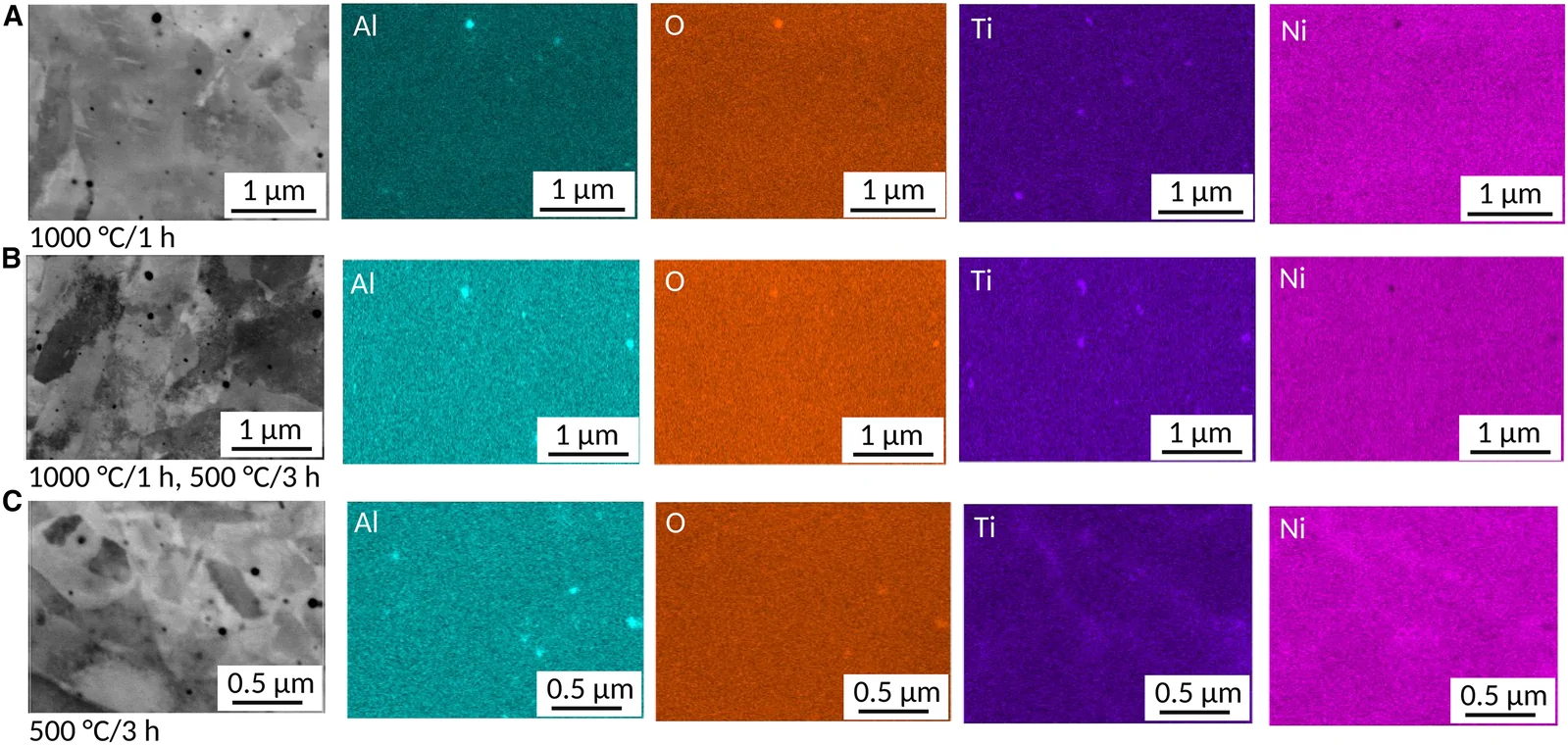
Heat treatment changes the distribution of oxide inclusions and Ti/Ni-enriched microstructural features in LPBF M789 BSE images and corresponding EDS maps of Al, O, Ti, and Ni are shown for (A) solution annealing at 1000°C for 1 h, (B) solution annealing at 1000°C for 1 h followed by aging at 500°C for 3 h, and (C) direct aging at 500°C for 3 h. Al/O-rich particles are present in all conditions, discrete Ti-rich particles are most evident in (A) and (B), and Ti/Ni enrichment along microstructural boundaries is most evident in (C). All images within each row were acquired from the same area. Scale bars, 1 μm in (A) and (B) and 0.5 μm in (C).

In both the AB and heat-treated conditions, small particles and chemically enriched features were observed in the microstructure (Figures 4 and 7). Based on the EDS maps, they are treated as two distinct populations rather than a single precipitate/oxide group. Al/O-rich particles are interpreted as oxide inclusions, consistent with partial oxidation of the powder or melt-pool material despite processing under a protective atmosphere. In contrast, Ti-rich particles, and Ti/Ni-enriched boundary particles, lacking clear oxygen co-localization, are described as local chemical heterogeneities associated with the LPBF/aging response. Samples subjected to solution annealing primarily contained discrete Ti-rich particles, whereas directly aged samples showed Ti/Ni enrichment along grain or cellular boundaries. A similar Ti/Ni segregation was also present in the AB condition, indicating that at least part of this heterogeneity is inherited from the AM process rather than formed solely during aging.

### Thermal stability

One anticipated application of this material is the production of molds for the pressure casting of non-ferrous metals (e.g., Zn and Al), where long-term thermal stress in the molds can reach approximately 350°C. Therefore, the thermal stability of additively manufactured and heat-treated M789 was also evaluated at this temperature. Samples were first solution-annealed and aged (1000°C/1 h, 500°C/3 h), and subsequently exposed in a furnace at 350°C in air (i.e., without a protective atmosphere) for 24 h, 160 h, 720 h, and 2880 h. The heat-treated sample without additional thermal exposure served as the reference (Table 3).

**Table 3:** Samples after heat-treatment 1000°C/1 h + 500°C/3 h, exposure in a furnace without a protective atmosphere at 350°C

	YTS [MPa]	UTS [MPa]	TE [%]	HV10 [-]	Oxide layer thickness [μm]
350°C/0 h	1610 ± 23	1735 ± 5	12 ± 1	558 ± 4	–
350°C/24 h	1625 ± 5	1737 ± 8	11 ± 1	562 ± 3	23.0 ± 3.7
350°C/160 h	1662 ± 6	1801 ± 8	8 ± 2	568 ± 4	20.3 ± 5.5
350°C/720 h	1686 ± 11	1868 ± 5	8 ± 3	577 ± 4	19.7 ± 3.8
350°C/2880 h	1772 ± 13	1891 ± 10	5 ± 2	593 ± 5	19.2 ± 4.4

Mechanical properties were measured using small flat samples. Oxide layer thickness was measured at cross-sections by image analysis.

Thermal exposure at 350°C for more than 24 h resulted in a gradual increase in tensile strength and hardness and a decrease in total elongation (Table 3). Yield strength first increased and then remained constant for a long time, increasing markedly again only after 2880 h of exposure at 350°C. The total change in mechanical properties, however, was only small, and only minor strengthening occurred. After 2880 h, the tensile strength increased by 156 MPa and the hardness by 35 HV10. The most remarkable was the decrease in total elongation, which fell to less than half of its original value. A one-way ANOVA across the five exposure times confirmed that all four measured properties differ significantly across the time axis (F(4, 20) ≥ 10.1, *p* < 0.001 for each). Welch’s pairwise tests with Bonferroni correction (m = 4) against the 0 h reference showed that the increase in ultimate tensile strength becomes significant from 160 h onward (corrected *p* ≤ 0.002), the increase in HV10 from 720 h (corrected *p* = 0.001), and the decrease in elongation from 2880 h (corrected *p* = 0.002), while yield strength shows a small transient increase at 24 h followed by a large significant increase at 720 h and beyond.

Quantitative image analysis of polished cross-sections showed that the oxide layer was already present after 24 h, with an average thickness of 23.0 ± 3.7 μm. Prolonged exposure did not lead to progressive thickening: the measured values were 20.3 ± 5.5 μm after 160 h, 19.7 ± 3.8 μm after 720 h, and 19.2 ± 4.4 μm after 2880 h. Because these values overlap within the scatter of individual measurements, the small apparent decrease should not be interpreted as physical thinning of the oxide scale. Instead, the results indicate that most of the measurable oxide-layer thickness developed during the first 24 h, whereas subsequent exposure mainly changed the internal chemistry and morphology of the scale.

**Figure 8:**
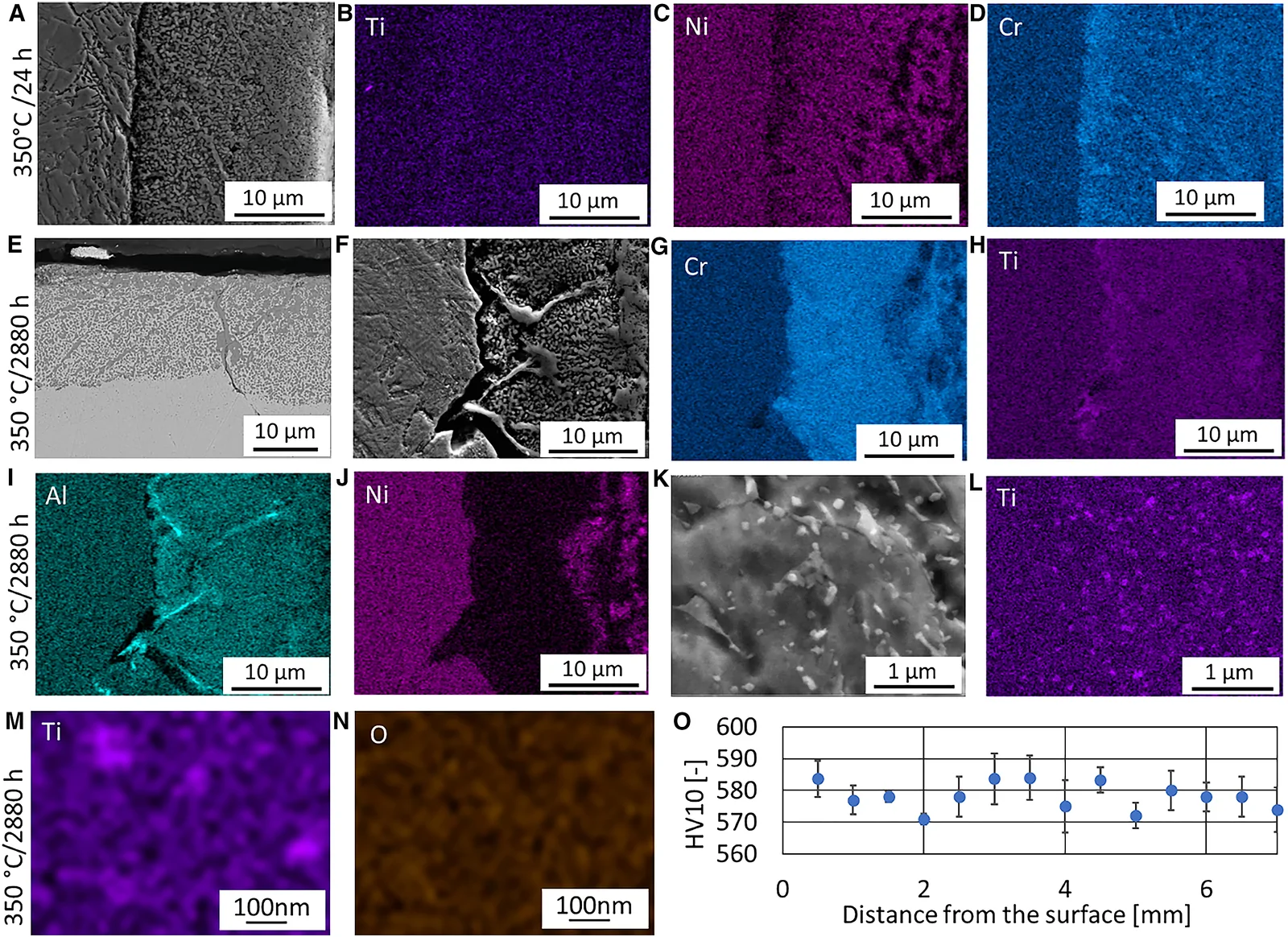
Long-term exposure at 350°C transforms the initially compact oxide into a chemically stratified scale and produces near-surface Ti-rich particles without a systematic hardness gradient (A) SEM image of the oxidized surface after 24 h and corresponding EDS maps of (B) Ti, (C) Ni, and (D) Cr. (E and F) SEM images after 2880 h and corresponding EDS maps of (G) Cr, (H) Ti, (I) Al, and (J) Ni. (K) BSE image of fine subsurface particles and (L) the corresponding Ti map. High-magnification EDS maps show (M) Ti enrichment in the particles and (N) the absence of co-localized oxygen. (O) HV10 hardness profile from the surface toward the sample center after 2880 h; error bars denote SD. Scale bars, 10 μm (A–J), 1 μm (K, L), and 100 nm (M, N).

After 24 h of exposure in air at 350°C, an oxide layer had already formed on the M789 steel surface (Figures 8A–8D). The layer appears relatively compact, showing only the first signs of chemical partitioning: the EDS maps indicate a localized Ni depletion at the metal/oxide interface (Figure 8C) together with Cr enrichment in the oxide compared with the matrix (Figure 8D), whereas Ti remains largely uniformly distributed at this length scale (Figure 8B). The metal/oxide interface is straight and no pronounced lateral segregation of the mapped alloying elements along the interface is observed. No obvious changes in the bulk microstructure are evident in the cross-section after 24 h exposure compared with the initial condition of the steel.

In contrast, long-term oxidation of M789 steel in air at 350°C for 2880 h led to pronounced development of both the oxide layer and the subsurface microstructure (Figures 8E–8L). Although the measured overall thickness of the oxidized surface layer changed only modestly between 24 h and 2880 h, its internal architecture changed substantially: the initially compact, weakly partitioned oxide developed into two compositionally distinct bands (Figures 8E–8J). This indicated pronounced selective oxidation of the alloying elements. EDS analysis revealed a bilayer structure consisting of an outer oxide layer enriched in Ni (Figure 8J) and Cr (Figure 8G) and an inner oxide layer adjacent to the metallic substrate, characterized by elevated Ti concentrations (Figure 8H) and highly localized enrichment of Al (Figure 8I), accompanied by a marked depletion of Ni (Figure 8J). Aluminum is selectively segregated within the inner oxide layer in the form of elongated, tongue-like features extending into the substrate (Figure 8I).

Although no matrix changes were resolved at the scale of the SEM observations, BSE images of polished cross-sections after 350°C/2880 h revealed very fine Ti-rich particles confined to the subsurface region beneath the oxide scale (Figures 8K and 8L). High-magnification EDS mapping showed that the oxygen signal was concentrated mainly in the oxide scale and was not co-localized with the Ti-rich particles (Figures 8M and 8N), indicating that the particles are not Ti oxides. No clear co-localized enrichment of Cr, Ni, Mo, or Si was detected at the same positions within the spatial resolution of SEM-EDS. Similar particles were not observed in the interior of the exposed block.

Over the same exposure series, mechanical testing showed a modest macroscopic change: after 350°C/2880 h, the ultimate tensile strength and HV10 hardness increased by 156 MPa and 35 HV10, respectively, while total elongation decreased from 12% to 5% (Table 3). Because the Ti-rich particles were confined to the near-surface region, and because the HV10 profile did not show a systematic hardness gradient across the section (Figure 8O), these bulk mechanical changes are not assigned to the observed subsurface particles. Instead, they are treated as the macroscopic response of the already aged metallic material to prolonged exposure at 350°C, whereas the oxide scale and subsurface Ti-rich particles are treated as a separate near-surface exposure response.

### Repair/redeposition feasibility

Hybrid parts were produced using a two-step AM procedure to simulate the repair of damaged AM components by subsequent material deposition. After building the first half of each cylindrical sample, the build plate was removed from the machine, the cylinder tops were ground to restore flatness and surface quality, and the plate was then returned to the printer for deposition of the second half, forming the hybrid sample.

### As-built hybrid condition

The printed samples were machined into mechanical test samples with a circular cross-section (Figure 2B). The tensile samples were designed such that the interface between the two build stages was located at the center of the gauge length. The results were then compared with those from samples produced in a single uninterrupted build (i.e., without removal from the machine, reinstallation, or subsequent deposition). The comparison shows that, for M789 steel, interrupting and resuming the AM process does not degrade performance: the mechanical properties of the hybrid parts are comparable to those of continuously printed samples (Table 4).

**Figure 9:**
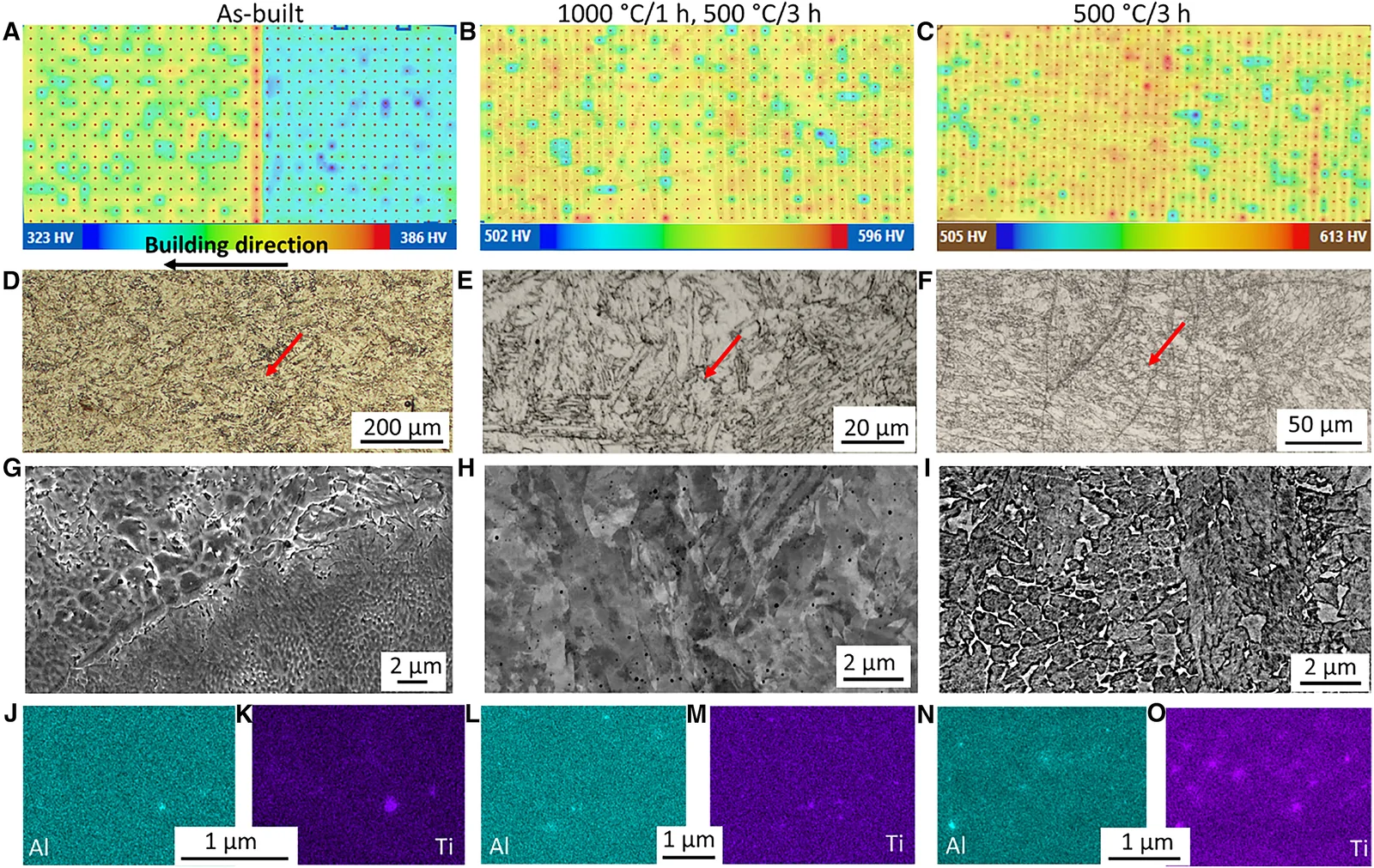
The redeposition interface remains microstructurally continuous after heat treatment, although local hardness variations persist Columns show the as-built hybrid condition (left), solution annealing at 1000°C for 1 h plus aging at 500°C for 3 h (middle), and direct aging at 500°C for 3 h (right). (A–C) HV0.1 hardness maps. (D–F) Light micrographs with the interface marked by a red arrow. (G–I) SEM details of the interface, acquired in SE mode in (G) and BSE mode in (H) and (I). (J–O) Corresponding EDS maps show Al- and Ti-enriched features. Scale bars, 200 μm (D), 20 μm (E), 50 μm (F), 2 μm (G–I), and 1 μm (J–O).

The bulk hardness of the AB M789 was around 317 ± 5 HV10. Hardness measured on the longitudinal section of the tensile-tested sample was higher in both locations, reaching 336 HV10 near the fracture and 343 HV10 at the opposite end, farthest from the fracture (Figure 9A). The fracture occurred within the region deposited during the second build step. The HV0.1 microhardness map (Figure 9A) reveals a distinct hardness discontinuity at the interface between the two build stages. The maximum value of 386 HV10 was recorded directly at the joint, and the hardened zone was relatively narrow. This local increase is attributed to thermal cycling during deposition of the second stage, which reheated the upper layers of the first stage, effectively serving as an *in situ* heat treatment. This is consistent with a local aging/precipitation response, expressed as fine Ti/Al-enriched particles in the transition region, thereby increasing hardness. On the fracture side of the sample, microhardness was slightly lower (blue) than on the side farther from the fracture (yellow-green-blue), consistent with the fact that failure occurred in the second-stage material.

The microstructure appeared similar on both sides of the interface. In optical micrographs, the transition was only faintly visible (Figure 9D) and became distinct mainly in SEM observations (Figure 9G). The subtlety of the interface is consistent with the absence of an increased density of AM-related defects at the joint between the two deposition stages.

### Heat-treated hybrid condition

The hybrid parts were subsequently heat-treated using (1) direct precipitation hardening at 500°C/3 h and (2) a two-step schedule consisting of solution annealing at 1000°C/1 h followed by precipitation hardening at 500°C/3 h. For each heat-treatment condition, three hybrid builds were prepared; from each, one large tensile sample was machined. Thus, the values reported in Table 5 represent averages of three measurements. In addition, the interface regions were examined microstructurally (Figure 9). For both heat-treatment routes, the hybrid samples exhibited an ultimate tensile strength approximately 100 MPa lower than that of the continuously printed counterparts, while the yield strength was reduced by 110–185 MPa. In contrast, hardness and ductility remained comparable between hybrid and uninterrupted builds.

#### Direct aging at 500°C/3 h

After direct precipitation hardening at 500°C/3 h, the interface between the two build stages remained discernible in the hardness map (Figure 9C) and in low-magnification overview images (Figure 9F). However, at higher magnification in SEM, the interface could no longer be clearly identified (Figure 9I), and the microstructure appeared as a uniform martensitic matrix. Ti- and Al-enriched particles were extremely fine, and they were identified primarily from EDS elemental maps (Figures 9N and 9O), which are shown only for elements exhibiting non-uniform distributions.

Similar Ti/Al-enriched particles were also observed in the heat-treated, continuously printed M789 samples discussed in the previous section. They are consistent with an aging-related precipitation response, but are not used here as direct SEM/EDS proof of η-Ni_3_(Ti, Al)-type precipitates. X-ray diffraction indicated approximately 5.8% retained austenite.

The microhardness profile across the transition exhibited substantial scatter, ranging from 490 to 630 HV0.1. The maximum values were recorded at the transition/junction region (Figure 9C). Cracking occurred in the material deposited during the second build step.

The directly aged hybrid condition (500°C/3 h) was selected for the detailed EBSD/KAM comparison because it represents the repair-relevant peak-strength condition and retained sufficient interfacial contrast after aging to allow the interface and the surrounding re-deposited material to be analyzed in the same specimen. The area far from the interface was taken from the same re-deposited material as an internal reference, so that local misorientation and apparent orientational grain size could be compared under identical preparation and EBSD acquisition conditions.

**Figure 10:**
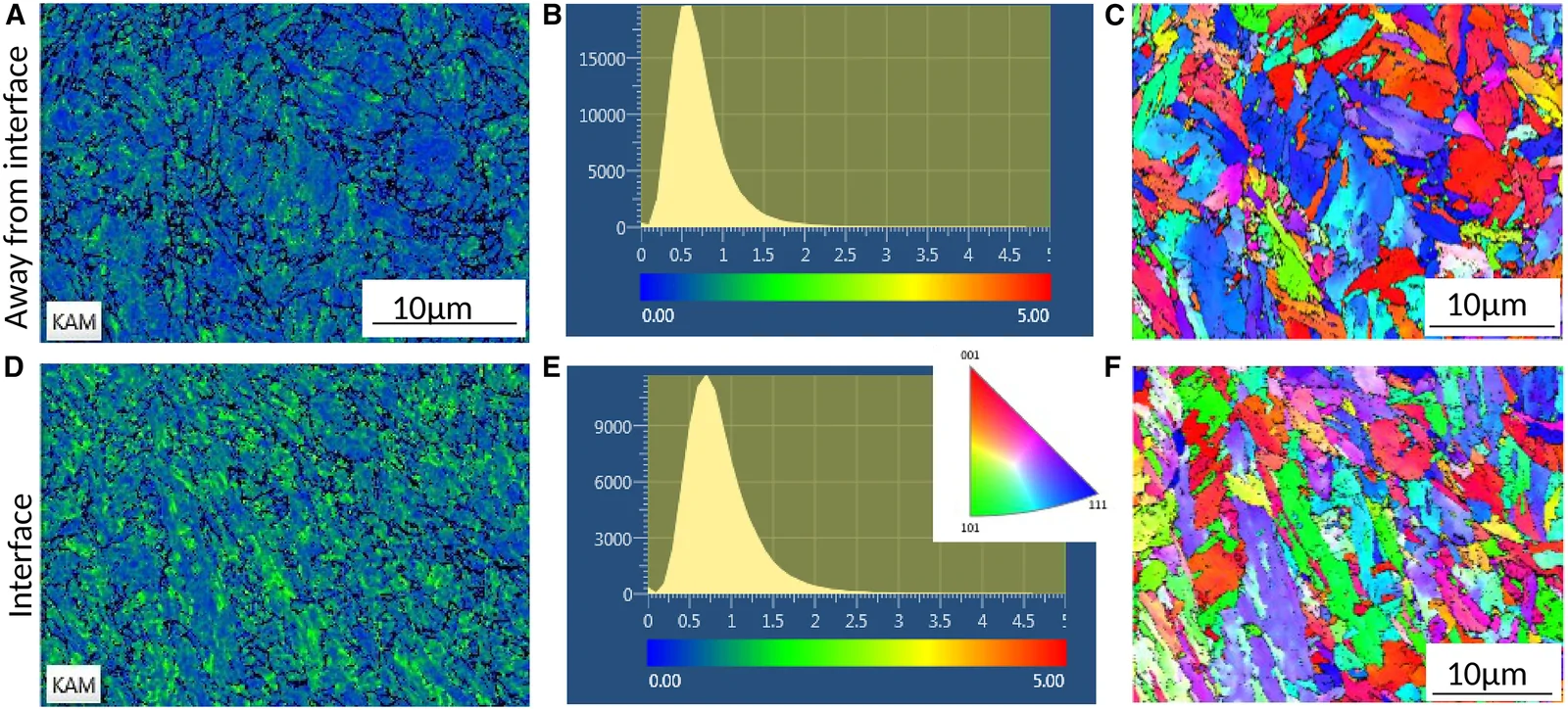
The directly aged redeposition interface exhibits more heterogeneous local misorientation but no pronounced crystallographic discontinuity or grain coarsening (A–C) Re-deposited material away from the interface: KAM map (A), KAM histogram (B), and IPF map (C). (D–F) Interface region: KAM map (D), KAM histogram (E), and IPF map (F). KAM values are shown on a 0–5° scale and were calculated using a 3 × 3 kernel and a 5° threshold. Scale bars, 10 μm (A, C, D, F).

The KAM maps show that the interface between the newly deposited M789 steel and the previously printed base is characterized by a more heterogeneous local misorientation distribution (Figure 10). The KAM values are given in degrees and displayed on a 0–5° scale, with a 3 × 3 kernel and a threshold angle of 5°. The same analysis parameter settings were used in both areas to ensure direct comparability of the results. Locally increased KAM values, visible as green bands and regions, are more pronounced near the interface, which may be associated with enhanced lattice deformation, residual strain, and microstructural heterogeneity caused by the redeposition process. In the newly deposited material far from the interface, the KAM distribution is dominated by lower values and appears more uniform, indicating a lower level of local misorientation compared with the interface region. The interface region contains a higher fraction of green regions and localized bands of increased KAM, indicating more pronounced orientation gradients and local strain heterogeneity. Away from the interface, the KAM values are generally lower and more uniformly distributed, suggesting a more homogeneous microstructural state of the material.

The IPF maps acquired near the interface and in the re-deposited material away from the interface revealed a fine, highly subdivided martensitic microstructure in both regions. No abrupt crystallographic discontinuity, coarse-grained interfacial zone, or preferentially oriented layer was observed at the transition between the original printed foundation and the subsequently deposited material. The interface region exhibited slightly greater local orientation contrast, whereas the material farther from the interface contained more elongated orientation domains. However, these differences were gradual and local in character. The EBSD-based orientational grain-size evaluation showed comparable values in the interface region and in the re-deposited material away from the interface. The mean apparent grain size was slightly higher near the interface (2.13 ± 1.54 μm) than farther from it (1.73 ± 1.69 μm), but the broad and strongly overlapping distributions indicate that this difference should not be interpreted as evidence of pronounced local grain coarsening. It should also be noted that these values were obtained from locally analyzed EBSD map areas and therefore represent supporting, rather than statistically exhaustive, grain-size information. In combination with the IPF and KAM maps, the results indicate that the re-deposition process did not produce a distinct, coarse-grained, or mechanically weak interfacial zone.

Although EBSD maps confirmed the presence of small retained-austenite islands, mainly along grain/lath boundaries, quantitative EBSD phase-fraction analysis was not used for regional comparison between the interface and the bulk of the re-deposited material. The retained austenite fraction was low, while the fraction of non-indexed points was considerably higher than the retained austenite fraction itself. Therefore, local EBSD-derived retained-austenite fractions would be highly sensitive to indexing quality and surface preparation and could not provide a reliable basis for correlating retained austenite with the local mechanical response.

#### Solution annealing and aging

After the two-step heat treatment, the interface between the two build stages was even less distinct than after direct precipitation hardening (Figures 9B–9E and 9H). Although the aging parameters were identical to those in the previous condition, the matrix morphology differed: the microstructure exhibited a more pronounced lath structure (Figures 9E–9H), formed during austenitization during the solution-annealing step. Fine dark particles were also observed at higher magnification (Figure 9H). EDS elemental maps (Figures 9L and 9M) again indicated local Ti and Al enrichment in these fine particles, consistent with an aging-related precipitation response, but not sufficient for direct crystallographic phase identification of η-Ni_3_(Ti, Al) precipitates. Nickel is the matrix-forming element of M789 and is therefore present throughout the field of view, including within the particles; the EDS maps in Figure 9 are shown only for elements with non-uniform spatial distributions (Ti, Al) for clarity, and the Ni signal does not provide additional contrast at the available resolution. XRD phase analysis showed a retained-austenite content of 5.6%, comparable to that in the directly aged condition (Figure 6M).

The microhardness profile across the interface showed less scatter than after direct aging, with values ranging from 500 to 585 HV0.1. For continuously printed samples subjected to the same two-step treatment, the hardness was around 564 HV10, which is in good agreement with the hybrid part.

## Discussion

In this work, LPBF-produced M789 maraging stainless steel was examined within a unified framework linking post-processing route, microstructural evolution, and mechanical performance. M789 is a relatively new alloy specifically designed for AM and, owing to its very low carbon content, exhibits excellent weldability, making it particularly well suited to laser-based processes. These attributes make it attractive not only for the fabrication of high-strength, corrosion-resistant tooling, but also for repair-oriented manufacturing concepts in which local remelting or redeposition must not compromise structural integrity. The heat-treatment space examined here was selected to broaden, rather than duplicate, the published LPBF M789 record. Prior LPBF M789 studies have predominantly explored the canonical 1000°C/1 h + 500°C/2–3 h route,^2^^,^^3^^,^^4^^,^^22^ with a single broader aging scan reported between 470°C and 590°C^23^ and isolated direct-aging investigations at 475°C–510°C.^2^^,^^24^ Of the 31 material states examined in the present work, 22 material states are not represented in prior LPBF M789 literature at all. Of the remaining 9 literature-overlapping states, 6 are only partially overlapping, because they fall within previously published aging windows or hardness maps but have not been reported as identical tensile-property states under the same processing/testing protocol. Only 3 states can be regarded as fully or near-fully represented by prior LPBF M789 studies. Material states not represented in the prior LPBF M789 literature, including, for example, 900°C and 1100°C solution-annealing temperatures, the 0.5 h and 1.5 h annealing hold times, and their combination with aging, and the long-term thermal exposure series at 350°C up to 2880 h. On the other hand, the 9 overlapping points correspond mainly to the canonical 1000°C/1 h anneal and aging in the 500°C–540°C/1–3 h region. In addition, LPBF redeposition onto previously printed M789 has not been previously documented, although hybrid M789-on-wrought-N709 builds have been examined.^14^ The contribution of the present work is therefore not the first observation of aging or solution treatment in M789, but the first internally consistent map across these combined processing routes, supplemented by long-term stability and same-material repair characterizations.

### Microstructure

In the present study, all tensile test samples were machined from AM material and subsequently surface-finished (cutting, grinding, polishing) to minimize roughness-related scatter. In practical components, however, such intensive post-processing is often impractical or economically unjustified. Consequently, the AB surface topography and near-surface defects—such as pores, oxides, and partially melted particles—may significantly affect service performance, particularly corrosion resistance and fatigue strength. Prior work has identified surface condition as a dominant factor governing fatigue behavior in LPBF parts.^5^^,^^20^ This has stimulated the development of in-process surface-improvement approaches, such as incremental dual-LPBF, in which each layer is produced with a high-power laser and subsequently remelted with a lower-power laser to enhance surface quality and densification.

A characteristic feature of additively manufactured M789 steel is the presence of retained austenite. In the present work, the microstructure comprised a predominantly martensitic matrix containing several per cent of retained austenite. Retained austenite was already detected in the gas-atomized powder, and its fraction decreased after LPBF due to the high cooling rates and repeated thermal cycling. In general, retained austenite lowers hardness and strength and can compromise long-term property stability, because delayed transformation to martensite during service may generate internal stresses and lead to distortion or dimensional changes.

Literature data indicate that approximately 6% of retained austenite is obtained after a 1000°C/1 h + 500°C/3 h treatment,^3^ increasing to around 8% with prolonged aging. In the present study, the same heat-treatment schedule produced 5.1–6.6% retained austenite, depending on the hold at 1000°C. The longest solution-annealing hold of 1.5 h resulted in the highest retained-austenite fraction of 6.6%. Direct aging at 500 °C/3 h resulted in 5.8% of retained austenite, comparable to the two-step schedules and supporting the view that direct aging can maintain a favorable strength-ductility balance.^17^

These observations are consistent with the precipitation and phase-transformation mechanisms reported in the literature, but the different particle populations must be separated. Al/O-rich particles observed in the AB and heat-treated conditions are treated as fine oxide inclusions. Ti-rich or Ti/Ni-enriched particles that are not clearly co-localized with oxygen are instead considered part of the aging-related chemical response of M789. Together with the pronounced increase in strength and hardness during aging and with prior TEM/APT studies, these observations support a precipitation-hardening interpretation. The specific assignment to η-Ni_3_Ti, η-Ni_3_(Ti, Al), Ni_3_(Mo, Ti), or related nanoscale phases is used as a literature-supported working description rather than as direct phase identification in the present specimens. Previous studies of aging in M789 report nanoscale plate-like and spherical precipitates, notably η-Ni_3_Ti and Ni_3_(Mo, Ti). The temperature range around 500°C represents the kinetic-thermodynamic window for Ni-rich Ni_3_(Ti, Al)/η-Ni_3_Ti-type precipitation in M789: Ni/Ti/Al lattice diffusion is sufficient for rapid precipitation, while precipitate dissolution, Ostwald ripening, and austenite reversion remain limited. For LPBF AMPO M789, Morri et al.^23^ reported peak hardness of 580–600 HV at 480°C–510°C across a 470°C–590°C × 0.25–16 h aging scan, with a hardening activation energy of 245–253 kJ mol^−1^ close to Ni diffusion in α-Fe, hardening below around 530°C and softening above 530°C consistent with the onset of austenite reversion and Ostwald-ripening; Tian et al.^4^ reported maximum hardness/strength at 450°C–500°C/2 h, with 500°C giving the highest η-Ni_3_Ti number density (around 1 × 10^24^ m^−3^) and no detectable reverted austenite. Analogous Cr–Ni–Ti maraging stainless grades show the same window: peak hardening at 480°C–510°C in Custom 465^24^^,^^25^ and a 450°C–500°C optimum in Fe–Cr–Ni stainless maraging steels analyzed by atom-probe tomography.^26^ Below this range, aging in practical times is incomplete; above 530°C–550°C, precipitate coarsening, partial dissolution of Ni_3_(Ti, Al), and reverted-austenite formation overtake precipitation strengthening, and the associated η-phase contribution to strengthening has been estimated at approximately 779 MPa for the canonical M789 condition.^14^^,^^22^ At higher aging temperatures or longer aging times, Ni is progressively released into the Fe matrix as Ni_3_(Mo, Ti) transforms to the more stable Fe_2_Mo, which promotes austenite reversion.^14^ Thermodynamic simulations (e.g.,^22^) show that the austenite fraction increases above about 500°C and that the alloy becomes fully austenitic at about 855°C. Increasing aging time at a given temperature also raises the amount of reverted austenite.^27^ Overall, η-Ni_3_(Ti, Al) precipitation primarily raises tensile strength, whereas austenite reversion tends to improve ductility at the expense of strength.^22^ Similar behavior has been reported for Ti-free grade 300 maraging steel, where higher aging temperatures increase reverted austenite and recrystallisation and reduce hardness,^28^ consistent with trends noted in.^22^ In the present work, however, aging at 500°C/3 h and 540°C/3 h produced similar amounts of retained austenite, suggesting that, within this relatively narrow temperature interval, the competing effects of precipitation and austenite reversion are only weakly affected by the modest temperature increase.

It should be emphasized that the present study does not aim to establish a quantitative precipitate-strengthening model for M789. Direct identification of η-Ni_3_Ti, η-Ni_3_(Ti, Al), Ni_3_(Mo, Ti) or related nanoscale phases would require TEM-based diffraction/chemical analysis or atom probe tomography. In the absence of such data, the precipitate assignment used here is intentionally conservative and literature-supported. The key conclusions of the present work do not rely on distinguishing between individual Ni-Ti-Al- or Ni-Mo-Ti-rich precipitate variants; they rely on the measured heat-treatment response, retained-austenite fractions, hardness, and tensile-property trends. Therefore, the term η-type or Ni-Ti-Al-rich precipitates is used as a working description of the expected aging-related strengthening phase family, rather than as direct confirmation of the phases present in the present specimens.

Compositional modifications can also tailor the fraction of retained austenite in M789. As shown in,^29^ alloying M789 with 316L steel alters austenite stability and can be used to tune the amount of retained austenite after solution annealing and aging, offering a potential route to optimize the strength-ductility balance.

### Mechanical properties

A summary of the mechanical properties of M789 steel after AM and heat treatment, obtained in this study, reported in the literature, and benchmarked against other maraging steels, is presented in Table 6. The manufacturer’s datasheet claims the highest properties for the combined treatment (1000°C/1 h + 500°C/3 h),^6^ reaching tensile strength of 1800–1900 MPa with 4–8% elongation; however, these values have not been consistently reproduced in published studies. For example, significantly lower strengths of 1610 MPa and ductility of 2% were reported in^3^ after the same heat treatment. This is attributed primarily to oxide inclusions rich in Ti and Al (Ti_2_O·Al_2_O_3_) located predominantly at grain boundaries and acting as crack initiation sites, as well as to a lower relative density, which indicates a higher pore content. In the present work, a strength of 1839 MPa and a hardness of 558 HV10 were obtained for the same treatment using the larger, circular cross-section samples, accompanied by an elongation of 3% (Table 5). These samples were selected for comparison because they more closely match standard tensile test geometries than the small flat samples and can therefore be compared more reliably (small flat samples generally provided lower tensile strengths and higher elongations). The measured yield and ultimate tensile strengths fall within the ranges specified in the datasheet, whereas elongation is marginally below the claimed threshold.

Published data for M789 aged at 500°C report a tensile strength of approximately 1790 MPa, an elongation of 9–11%, and a hardness of approximately 52 HRC,^4^ which is in good agreement with the present results obtained using small flat samples, where tensile strength reached 1767 MPa, a hardness of 53 HRC and an elongation of 12% (Figure 5).

Overall, the results confirm that sample size and geometry exert a measurable influence on the tensile response and should be considered when comparing M789 data across conditions and with the literature. Because the geometry-induced offset is systematic, the absolute values obtained from small flat and round specimens should not be directly interchanged. Nevertheless, this does not affect the interpretation of heat-treatment trends, because each comparison is made within a single specimen geometry and relative to its own reference condition. To avoid ambiguity, we note explicitly which geometry underlies each reported value. The temperature × time heat-treatment scan in Figure 5 and the long-term thermal-stability data in Table 3 were obtained on small flat samples, chosen to minimize material consumption across the wide aging window. The hybrid-deposition comparison in Table 5 and the cross-grade benchmarking in Table 6 used the larger round samples to match standard tensile-test geometries for cross-study comparison. The AB reference is reported for both geometries in Table 2. Because the geometry-induced offset (small-flat yield strength systematically lower by approximately 80–120 MPa and elongation higher by 5–15 percentage points; see Tables 2 and 4) is systematic, comparisons within a single geometry remain internally consistent, and absolute values can be reconciled via the offset reported here. Direct cross-geometry comparison without this caveat is not appropriate, and the present work does not report it. Small flat specimens in this work generally produced slightly lower strengths and notably higher elongations than the larger circular specimens, which indicates that specimen geometry should be considered when comparing data across conditions and studies.

The difference in elongation is expected because elongation is particularly sensitive to gauge length and constraint. Shorter gauge lengths typically yield higher percentage elongation because localized necking contributes more strongly to the measured strain, whereas longer gauge lengths reduce the reported total elongation. In addition, thin, flat specimens deform under lower through-thickness constraints and approach a plane-stress condition. This reduces stress triaxiality in the necked region and increases the strain to crack initiation. In contrast, axisymmetric round specimens exhibit a more constrained stress state in the neck, with higher triaxial stress, which promotes earlier damage evolution and reduces the measured elongation.^30^^,^^31^

Although yield and ultimate tensile strengths are often treated as geometry-independent material properties, small flat mini-samples systematically reported slightly lower values than larger round samples in this work (for example, 1735 MPa vs. 1839 MPa for two-step heat-treated samples 1000°C/1 h + 500°C/3 h). The most plausible explanation is that this offset is dominated by experimental and geometric factors and by increasing sensitivity to non-idealities with decreasing cross-section, rather than by an intrinsic difference in the heat-treated M789 microstructure, as the same effect was also reported for AM maraging steel 18Ni-300^32^. First, axiality/alignment matters more for smaller samples. ASTM E8/E8M explicitly warns that any departure of the sample axis from the load train introduces bending stresses that are not captured by the usual stress calculation, and it quantifies that a given eccentricity produces a larger stress error as sample diameter decreases.^33^ Second, small flat samples have a higher surface/edge-to-volume ratio, so the effective load-bearing area is more influenced by edge rounding, surface roughness, and cutting/finishing quality.^34^ Related experimental work on miniaturized tensile testing shows that measured yield and ultimate tensile strengths can differ by several per cent, up to 10–15%, when comparing standard and increasingly miniaturized geometries,^31^ consistent with the offsets observed in the present work. The influence of specimen geometry on measured tensile properties is a well-documented phenomenon for metallic materials in general^32^^,^^33^^,^^35^ and for AM maraging steels specifically^32^; the contribution of the present work is not to re-establish this effect but to quantify and explicitly bound its magnitude for LPBF M789 within a single internally consistent dataset, so that future cross-study comparisons can be placed on a defensible footing.

The yield-strength standard deviations on the larger round AB samples were 108 MPa in the X and Y directions and 115 MPa in the Z direction, and were therefore notably wider than those measured on the small flat samples (20–26 MPa; Table 2). The Z-direction value of 115 MPa is the value used for the continuously printed reference in Tables 4 and 6. This scatter is retained as part of the measured response rather than treated as an outlier. The magnitude of the scatter is also consistent with behavior reported for additively manufactured maraging steels when tested gauge-volume samples heterogeneous defect populations or spatially varying thermal histories. For 18MAR300, Tillmann et al.^36^ reported tensile-strength standard deviations of 68 MPa for AB LPBF material and 116 MPa for heat-treated LPBF/LPBF joints, and attributed the variability to sample-to-sample differences in interlayer defects. In DED (direct energy deposition) 18Ni300, a wide yield-strength dispersion was similarly associated with microstructural heterogeneity arising from repeated remelting and reheating of successive tracks and layers.^37^ In M789 specifically, Ti/Al-rich oxide inclusions and secondary porosity have been identified as metallurgical defects that can affect plasticity and crack initiation.^3^ The present 108–115 MPa scatter in YTS is therefore interpreted as a measured feature of the larger AB round specimens, most plausibly associated with the onset of plastic deformation in a heterogeneous LPBF microstructure. Importantly, the scatter is largely confined to the 0.2% proof/yield strength, whereas UTS and total elongation remain tightly grouped, indicating that the variability affects early yielding rather than final tensile failure.

For comparison, 18Ni-300 maraging steel, which is one of the most established maraging alloys in AM, typically exhibits substantially higher tensile strength of 2.2 GPa, yield strength of 2.0 GPa, and hardness of 650 HV after aging at 490°C, but at the expense of very low ductility around 1.6%.^9^ A further limitation of 18Ni-300 is its comparatively poor corrosion resistance. M789, therefore, represents a more balanced alternative. As shown in Table 6, M789 in the aged condition reaches slightly lower strength and hardness than 18Ni-300, yet remains significantly stronger than standard stainless steels such as 316L,^35^^,^^38^ while simultaneously offering markedly improved corrosion resistance compared with 18Ni-300 maraging steels.^39^

Toughness behavior further highlights the differences between heat-treatment regimes. According to,^1^ M789 combines the strength level of W722 AMPO with the corrosion resistance of 17-4 PH, and after the two-step treatment (1000°C/1 h + 500°C/3 h), it reportedly achieves higher impact toughness (17 J) than W722. In the present work, instrumented bending impact tests yielded toughness values of 8 J for the two-step regime and 11 J for direct aging at 500°C/3 h. By contrast, the AB condition showed much higher absorbed energies: approximately 135 J in the Z direction, 132 J in the Y direction, and 116 J in the X direction. These values are relatively high but consistent with the higher ductility measured in the AB state. The pronounced loss of toughness after aging reflects the change from a comparatively soft, ductile martensitic matrix to a precipitation-strengthened microstructure with reduced capacity for plastic deformation.

**Figure 11:**
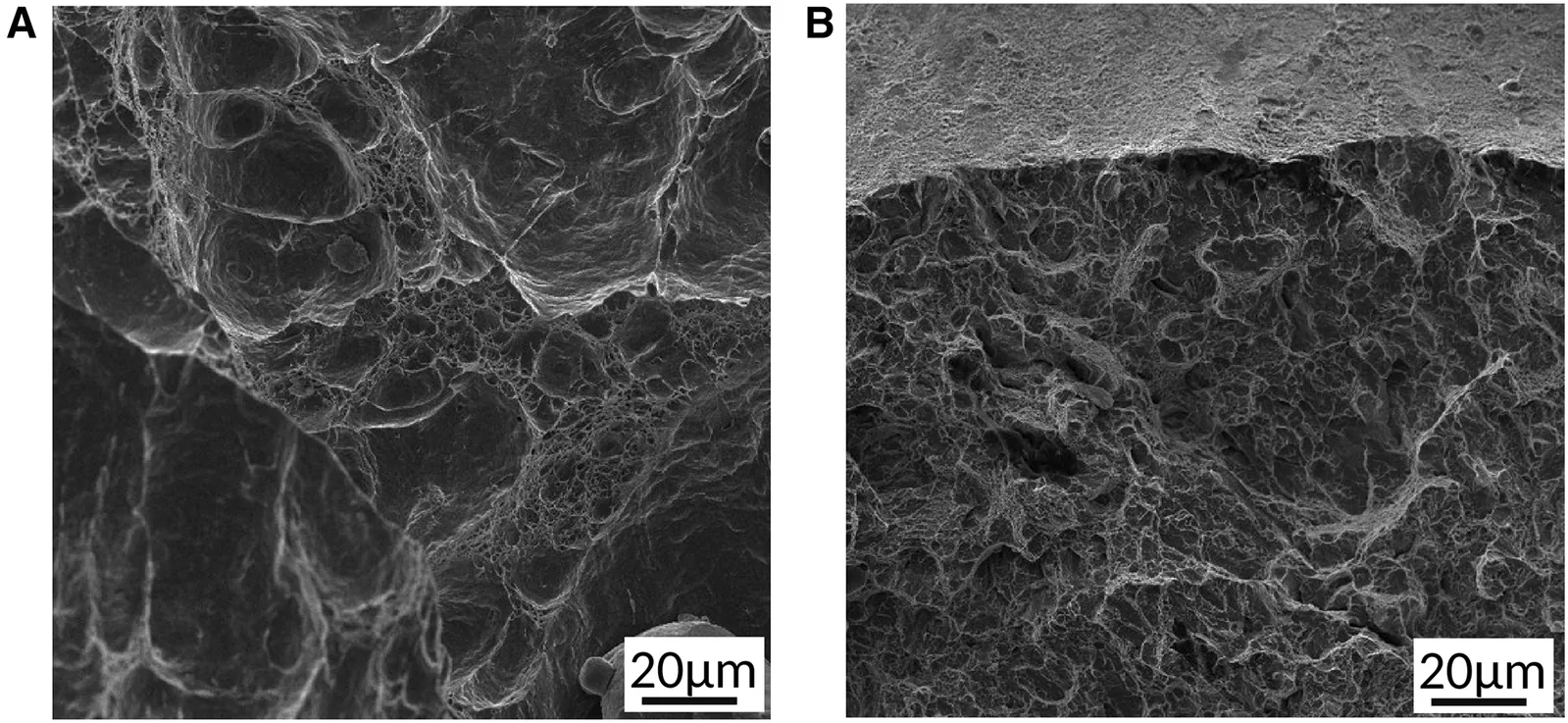
Aging changes the impact-fracture morphology from predominantly ductile to mixed mode Representative fracture surfaces after Charpy V-notch testing of (A) the as-built condition and (B) the directly aged condition (500°C for 3 h). Scale bars, 20 μm (A and B).

The fracture surfaces after the bending impact tests (Figure 11) illustrate these changes. In the AB condition (Figure 11A), the fracture surface is predominantly ductile with extensive plastic deformation and a high density of dimples, consistent with the high absorbed energy and elongation. An unmelted powder particle is visible in the lower-right area, which is typical of LPBF parts and results from insufficient local remelting; it is difficult to eliminate entirely. After direct aging (Figure 11B), the fracture surface changes to a mixed-mode character, with both ductile and brittle regions, consistent with reduced ductility and toughness. In agreement with this trend,^3^ reported an impact toughness of 11 J after 1000°C/1 h + 500°C/3 h and a considerably higher value of 21 J after solution annealing alone, further confirming the embrittling effect associated with extensive precipitation during aging.

The main advantage of M789 over conventional maraging steels is its improved corrosion resistance. Although its aged strength and hardness are slightly lower than those of 18Ni-300, they remain well above those of standard stainless steels such as 316L.^35^ Previous works have assessed corrosion behavior mainly in the AB condition and after the two-step treatment,^5^ concluding that surface condition and near-surface discontinuities (pores, oxides, partially melted particles) strongly influence corrosion response and can act as preferred sites for localized attack.

Taken together, the present mechanical and microstructural results, combined with these corrosion findings, indicate that direct aging without prior solution annealing is a promising heat-treatment route for AM M789. A limitation of the present microstructural assessment is that the nanoscale strengthening phase family responsible for aging hardening was not directly resolved or crystallographically identified. The supporting evidence in this work comprises: first, EDS-detected Ti and Al enrichment in fine particles, second, XRD-quantified retained-austenite fractions consistent with literature values for analogous heat-treatment schedules, and third, mechanical responses (peak strength at 500°C/3 h, with over-aging beyond 540°C) that follow the kinetic signature of η-type precipitation reported elsewhere.^4^^,^^14^^,^^22^ M789 delivers very high strength and hardness with relatively good ductility, a controlled amount of retained austenite, and does not appear to compromise corrosion performance. Nevertheless, this assessment should be validated under service-relevant conditions, particularly with respect to localized corrosion under realistic surface states, as well as wear behavior, given the intended applications of M789 in tooling, molds, and other highly loaded additively manufactured components.

### Thermal stability during exposure

Long-term oxidation of M789 steel in air at 350°C resulted in the formation of two oxide layers, arising from pronounced selective oxidation of the alloying elements and diffusion-controlled scale growth.^40^^,^^41^ EDS analysis revealed an outer oxide layer enriched in Ni and Cr, and an inner oxide layer adjacent to the metallic substrate, characterized by elevated Ti concentrations and a highly localized enrichment of Al, accompanied by a marked depletion of Ni. The development of this layered oxide scale can be explained within the framework of Wagner’s theory of selective oxidation, which describes the competition between external and internal oxidation as governed by oxygen permeability of the growing oxide scale and the relative activities and diffusivities of the alloying elements.^41^^,^^42^ In the present case, the high thermodynamic affinity of Al and Ti for oxygen, combined with their low diffusivity, favors internal oxidation near the metal/oxide interface rather than the formation of a continuous external Al_2_O_3_ or TiO_2_ scale.^40^^,^^41^^,^^43^ Consequently, Ti and Al oxidize predominantly *in situ*, giving rise to an inner oxidation zone composed mainly of TiO_2_ containing finely dispersed Al_2_O_3_. The absence of a continuous, protective oxide layer allows sustained oxygen ingress, thereby maintaining the conditions required for internal oxidation, as predicted by Wagner’s model.^41^^,^^42^^,^^43^^,^^44^^,^^45^ Wagner’s transition criterion locates the boundary between internal oxidation and an external continuous oxide of a reactive solute B at a critical bulk fraction N_Bˆcrit proportional to (N_Oˆ(s) · DO/DB)ˆ(1/2), where N_Oˆ(s) is the oxygen activity at the alloy/scale interface and DO/DB is the ratio of oxygen to solute lattice diffusivity. For M789 at 350°C (623 K), treating the martensitic matrix as α-Fe for a first-order estimate, literature-extrapolated Arrhenius coefficients give DO ≈ 2 × 10^−14^ m^2^ s^−1^ for interstitial oxygen. At the same time, solute lattice diffusivities are DNi ≈2 × 10^−25^, DTi ≈6 × 10^−26^, DAl ≈4 × 10^−30^, and DCr in the range 10^−28^–10^−25^ m^2^ s^−1^ depending on the ferromagnetic correction.^46^^,^^47^^,^^48^ The resulting ratios DO/Dmetal of 10^11^–10^16^ place dilute Al and Ti (≈1 at.%) firmly on the internal-oxidation side of the Wagner criterion, whereas the much higher Cr concentration (≈12 at.%) supports outward participation in a thin Cr-bearing external layer. This kinetic asymmetry explains why Al and Ti are not observed as a continuous external alumina/titania scale but instead appear in the inner oxide and as tongue-like internal-oxidation features along grain and cell boundaries, while Cr and Ni — the latter benefiting from rapid grain-boundary transport in Cr-rich oxide scales39 — concentrate in the outer band. The apparent saturation of overall scale thickness is also consistent with parabolic, diffusion-controlled growth: for a small low-temperature rate constant kp ≈ 10^−21^–10^−20^ m^2^ s^−1^, the predicted increment over 2880 h is only 0.1–0.3 μm, below the resolution of routine cross-sectional microscopy, while compositional rearrangement within the scale continues without significant net thickening.^41^

Nickel, whose oxide exhibits lower thermodynamic stability and which possesses significantly higher diffusion mobility, is progressively displaced toward the surface during oxide growth and contributes primarily to the formation of the outer Ni-rich oxide layer. Chromium participates in oxidation throughout the scale, probably forming solid solutions within both Ti- and Ni-rich oxides; however, at the relatively low temperature of 350°C, the kinetics of Cr_2_O_3_ formation are insufficient to establish a continuous protective layer.^45^^,^^49^ A characteristic feature of the inner oxide layer is the highly selective segregation of aluminum in the form of elongated, tongue-like features extending from the metal/oxide interface into the substrate. These features are interpreted as products of internal oxidation along fast oxygen diffusion paths, such as grain boundaries or defect-rich regions, where oxygen penetration is locally enhanced.^41^^,^^49^ At intermediate temperatures, chemically stratified, multi-layer nanometric oxides with steep concentration gradients are commonly reported, even during early oxidation of Cr-bearing alloys, supporting the observed compositional separation at this scale.^50^

Long-term exposure at 350°C for 2880 h affected the material in two distinct ways: it promoted aging-like changes in the metallic matrix, which were reflected in the measured mechanical properties, and it also led to the formation of a chemically stratified oxide scale at the surface. These two effects should be considered separately. Compared with the reference condition (1000°C/1 h + 500°C/3 h, without further exposure), the ultimate tensile strength increased by about 156 MPa and the hardness by roughly 35 HV10 after 2880 h at 350°C, while the yield strength remained virtually unchanged up to 720 h and increased significantly only after the longest exposure time. These changes indicate that prolonged exposure promoted additional aging-like strengthening of the metallic matrix. The present SEM-EDS and XRD data, however, do not identify the specific precipitate population responsible for this change. The exposed surface developed a chemically stratified oxide scale and local subsurface Ti-rich particles beneath it. Because these Ti-rich particles were confined to the near-surface region, were not observed in the interior, and were not accompanied by a measurable hardness gradient across the section, they are not treated as the origin of the bulk tensile response.

In contrast to the slight strengthening, the total elongation decreased more markedly, falling to less than one-half of its initial value after 2880 h. This reduction in ductility is also interpreted as a consequence of aging-like microstructural changes in the metallic matrix, particularly additional precipitation in the martensitic structure, which can reduce strain-hardening capacity and accelerate damage accumulation during tensile loading. The oxide layer formed during exposure represents a separate surface degradation phenomenon associated with oxidation in air. Under the tensile-testing conditions used in this study, the oxide scale is not considered to control the measured strength, hardness, or ductility values; rather, it provides complementary information on the surface stability of M789 during long-term exposure at 350°C.

Although the oxide scale thickness did not visibly increase between 24 h and 2880 h, its chemical stratification demonstrates that selective oxidation continued during long-term exposure. This is relevant mainly for surface-sensitive service properties, such as corrosion resistance, fatigue crack initiation, wear, or thermal-fatigue behavior, rather than for the monotonic tensile properties reported here. Therefore, the mechanical-property evolution and oxide-scale formation are treated as two parallel consequences of prolonged exposure at 350°C: the former reflects aging-like changes within the metallic material, whereas the latter reflects the oxidation response of the exposed surface. This distinction also underlines the potential benefit of surface protection, such as coatings or controlled atmospheres, in applications involving prolonged exposure to air. From an application perspective, the observed evolution is favorable. Yield strength remains high, and the moderate increases in tensile strength and hardness indicate that detrimental over-tempering does not occur at 350°C, even after several thousand hours. It should be emphasized that the present 350°C exposure series was designed to characterize long-term thermal stability of a pre-optimized heat-treatment condition (1000°C/1 h + 500°C/3 h), not to optimize an additional service-temperature treatment. Within this scope, the data in Table 3 serve as a property-vs-exposure-time map, allowing designers to estimate degradation for any specified service duration. These results, however, should not be interpreted as a general parametric description of the combined effects of heat-treatment route, exposure duration, oxidation behavior, precipitation evolution, and mechanical properties. Establishing such relationships would require a separate systematic study involving several heat-treatment conditions and exposure times.

The reduction in ductility, while significant in relative terms, remains within ranges typical of strongly precipitation-hardened tool steels and is unlikely to be the dominant limiting factor in service dominated by fatigue and thermal shock.

### Repair/redeposition feasibility

The final part of the study addressed the feasibility of repairing damaged or worn additively manufactured components by depositing M789 onto previously printed material. Hybrid cylindrical samples produced in two AM steps were used to simulate a practical repair/redeposition procedure. It is important to note that this route does not represent an isolated process-interruption experiment. Instead, it combines interruption, removal, and reinsertion of the build plate, grinding of the previously printed surface, possible modification of surface residual stresses and surface chemistry, and local reheating during the second deposition step. In the AB condition, the comparison between continuously printed and hybrid samples nevertheless indicates that this complete repair route did not introduce a critical weak plane: tensile strength was practically identical, elongation overlapped within experimental scatter, and the slightly lower yield strength of the hybrid parts was small relative to the scatter.

Microhardness mapping revealed a narrow band of increased hardness at the interface between the first and second print. This local hardening can be explained by reheating the upper layers of the first build during deposition of the second, effectively serving as a short local heat treatment that may promote additional local aging/precipitation. Notably, a tensile fracture occurred in the material printed in the second step rather than at the interface, confirming that the joint is mechanically sound. The microstructure remained martensitic in both parts, and no elevated concentration of lack-of-fusion defects or pores was observed at the interface.

After heat-treatment, the behavior of the hybrid parts remains mainly satisfactory, although some differences relative to continuously printed reference samples become more pronounced. Welch’s two-sample t-tests confirm that the strength reductions are statistically significant: for the 500°C/3 h route, yield strength decreased by 117 MPa (t = +5.27, df = 3.8, *p* = 0.007) and ultimate tensile strength by 101 MPa (*p* = 0.004); for the 1000°C/1 h + 500°C/3 h route, yield strength decreased by 167 MPa (*p* = 0.002) and the 110 MPa ultimate-strength reduction was marginal (*p* = 0.068). Differences in elongation and hardness between hybrid and continuously printed parts were not statistically significant in either route. These strength reductions should therefore be interpreted as the response of the complete repair/redeposition route rather than as a direct measure of intrinsic interface strength. The experiment does not separate the effects of interruption, grinding, surface reconditioning, residual-stress modification, possible oxidation or contamination of the prepared surface, realignment, and the *in situ* thermal cycle during redeposition. For both precipitation-hardening regimes investigated, the hybrid samples exhibited ultimate tensile strengths approximately 100 MPa lower and yield strengths 110–185 MPa lower than their continuously printed counterparts. At the same time, hardness and ductility were comparable. This combination suggests that global precipitation strengthening and matrix hardness were similar, whereas subtle differences in residual stress state, dislocation density, local precipitate distribution, defect population, or thermal history of the second build may reduce the macroscopic yield stress without producing a weak interface.

After direct aging at 500°C, the interface remains visible only at low magnification; SEM images show a homogeneous martensitic matrix with fine Ti/Al-enriched features detectable mainly through EDS contrast. These features are consistent with an aging-related precipitation response. X-ray diffraction confirms a similar retained austenite fraction around 6% as in the continuously printed material. The microhardness profile across the interface shows greater scatter (490–630 HV0.1) and a local maximum at the joint, reflecting subtle precipitation inhomogeneities and possibly residual stresses from the complex thermal cycle. Nevertheless, fractures still initiated in the second-printed region and not along the joint, which again indicates that the interface is not the weakest link.

After the two-step heat-treatment (1000°C/1 h + 500°C/3 h), the interface becomes even less distinguishable microstructurally. The lath martensitic morphology that forms during austenitization is continuous across the former joint, and Ti/Al-enriched fine features are distributed across both regions. The retained austenite content, around 6%, is essentially unchanged from the directly aged condition and the reference material. In this state, the microhardness variation across the interface is reduced to 500–585 HV0.1, indicating a more homogeneous microstructure. The remaining decrease in global yield and tensile strength of the hybrid parts is therefore likely not caused by a weak or defective interface, but rather by subtle differences in the overall thermal and mechanical history (e.g., slight misalignment, surface preparation before the second print, or differences in porosity level) that cannot be easily resolved by microscopy.

From an application perspective, the reductions in strength observed for the repaired hybrid parts after heat treatment remain relatively modest. For many tooling and mold applications, this penalty may be acceptable, particularly because hardness and ductility remained comparable to those of continuously printed material, and the repaired interface did not act as the preferred fracture path. These findings support the feasibility of the complete LPBF-based repair/redeposition route for M789, but they should not be interpreted as proving that the interface alone caused the measured strength reduction. Isolating the individual contributions of process interruption, grinding-induced surface state and residual stress, removal/reinsertion alignment, local oxide formation, and redeposition thermal history would require additional control experiments, such as interrupted builds without removal from the chamber, interrupted-and-reinserted builds without redeposition, builds with controlled surface preparations, and local interface-specific mechanical tests. Such a factorial study is beyond the scope of the present work, which focuses on application-oriented repair feasibility. Future work should therefore address both mechanistic control experiments for the repair interface and, in particular, the fatigue and thermal-fatigue behavior of repaired structures, as these properties will ultimately determine service reliability in real tooling applications. For the same reasons, intrinsic comparison of fatigue performance between Co-free M789 and Co-containing 18Ni-300 cannot be drawn from existing literature alone: reported LPBF M789 R = 0.1 HCF strengths range from 122 to 165 MPa for as-printed specimens to 192–282 MPa for polished specimens, and can be raised further by in-process surface improvement,^5^^,^^21^^,^^51^ while LPBF 18Ni-300 spans 178–600 MPa depending on heat treatment, surface finish, and residual-stress state.^52^^,^^53^ In both alloys, fatigue is dominated by surface roughness, lack-of-fusion defects, pores, and Ti/Al-rich oxide inclusions rather than by intrinsic alloy chemistry, and the available datasets differ in stress ratio, geometry, and surface state. A controlled, matched-condition fatigue comparison is therefore needed; fatigue characterization is outside the scope of the present work.

### Property-based design guidelines

The internally consistent dataset generated in this study allows the formulation of explicit post-processing guidelines linking heat-treatment route to expected mechanical performance, thermal-exposure tolerance, and repair compatibility. Rather than recommending a single “best” treatment, property-driven selection, in which the designer specifies the dominant service requirement and reads off the recommended route from the dataset assembled in Tables 2, 3, 4, and 5, is proposed.

Step 1 — identify the dominant property requirement. Tooling applications differ substantially: high-pressure die-casting molds prioritize strength and thermal-exposure tolerance; injection-molding inserts emphasize hardness and wear resistance; repaired tooling additionally requires interface integrity. The four post-processing routes considered span the practical design space: AB (Table 2), direct aging (DA, 500°C–520°C/3 h; Figure 5; Table 5), solution annealing only (SA, 1000°C/1 h; Figure 5), and the two-step solution + aging route (SA + DA, 1000°C/1 h + 500°C/3 h; Table 5).

Step 2 — match the requirement to route. Peak strength and hardness are obtained with DA on a continuously printed part (YTS = 1789 MPa, UTS = 1872 MPa, HV10 = 562; Table 5), with SA + DA providing comparable values (YTS = 1742 MPa, UTS = 1839 MPa) at the cost of slightly lower ductility. Maximum impact toughness is found in the AB or SA conditions (130 J; the heat-treated samples section), at the expense of yield strength below 800 MPa. SA alone offers no clear mechanical advantage over AB but produces a more homogeneous microstructure and is therefore reserved either as a pre-aging step or for applications where build-direction effects must be removed.

Step 3 — apply thermal-exposure correction (if applicable). For service at around 350°C in air, Table 3 provides a property-vs-time map. As a first-order rule, designers should reduce the allowable design strain by approximately a factor of 2 relative to the as-aged condition for service durations beyond 720 h at 350°C, while maintaining or improving peak strength margins. Surface protection (coatings, controlled atmosphere) is recommended for components for which the brittle oxidized surface layer would otherwise govern fatigue or impact response.

Step 4 — apply repair-condition correction (if applicable). For hybrid LPBF-repaired parts after the same heat treatment, the achievable yield strength is approximately 110–185 MPa lower and the ultimate strength approximately 100 MPa lower than for continuously printed monolithic counterparts (Welch’s *t* test, *p* ≤ 0.01 for yield strength; Table 5). Hardness and elongation are statistically indistinguishable.

The interface itself was not the weakest path, because fracture consistently initiated in the second-stage deposit rather than along the joint. However, the strength reduction should be treated as a correction for the complete repair/redeposition route, not as an isolated interface-strength penalty. It may include contributions from surface grinding, grinding-induced residual stresses, local surface oxidation or contamination before redeposition, realignment, and the thermal history of the second build. A conservative design practice is therefore to derate the yield strength of the repaired region by approximately 10% relative to the monolithic datasheet value unless repair-specific qualification tests are available.

These guidelines convert the heat-treatment map and its associated thermal-exposure and repair characterizations into actionable design rules, addressing the principal use case for which M789 was originally introduced — durable and repairable tooling for thermally and mechanically demanding service.

### Conclusion

LPBF enabled the production of dense M789 components with a predominantly martensitic microstructure containing a small fraction of retained austenite, providing a robust baseline for subsequent property tailoring by heat treatment. Across the post-processing space investigated here, heat treatment emerged as the key design variable governing the balance between strength, hardness, ductility, thermal stability, and repairability. Direct aging and combined solution annealing plus aging produced pronounced precipitation strengthening consistent with the expected aging response of M789, whereas solution annealing alone primarily homogenized the microstructure without delivering a clear mechanical advantage.

Peak strength and hardness were achieved by direct aging at 500°C–520°C for 3 h, yielding ultimate tensile strengths of up to 1.87 GPa and hardness values of 562–572 HV10. A conventional two-step route, comprising solution treatment at 1000°C for 0.5–1.5 h followed by aging at 500°C for 3 h, provided comparable strength and hardness together with retained austenite levels of 5–7%, indicating that the additional solution step is not essential when maximizing strength is the primary objective. At the same time, the pronounced loss in toughness under peak-aging conditions reveals a critical strength-toughness trade-off that must be considered in tooling design. The comparison of two tensile specimen geometries further showed that ductility is geometry-sensitive and should therefore be interpreted with caution when comparing datasets across studies or against datasheet values.

Prolonged exposure at 350°C for up to 2880 h caused modest macroscopic strengthening and reduced ductility, consistent with continued aging-like evolution of the already heat-treated matrix. Separately, exposure in air produced a chemically stratified oxide scale and local subsurface Ti-rich particles; these near-surface features are not treated as the origin of the bulk tensile response. Finally, LPBF redeposition onto previously printed M789 was feasible as a repair strategy, and the interface remained metallurgically sound. It did not become the preferred fracture path, while heat-treated hybrid parts retained comparable hardness and ductility despite moderate reductions in yield and ultimate strength. These strength reductions are therefore interpreted as a response of the combined repair route rather than as evidence of an intrinsically weak interface. Taken together, these findings move beyond a material-specific optimization study and establish a property-based framework for balancing peak strength, toughness, thermal-exposure tolerance, and repairability in LPBF-produced M789. More broadly, they provide practical guidance for designing durable, repairable maraging stainless steel tooling for demanding thermal and mechanical service conditions.

### Limitations of the study

Several limitations should be considered when interpreting the results. The nanoscale precipitates responsible for aging hardening were inferred from indirect evidence, including the heat-treatment-dependent mechanical response, SEM/EDS-detected Ti/Al enrichment, XRD-derived retained-austenite fractions, and comparison with prior M789 literature. They were not directly resolved or phase-identified by TEM, TEM diffraction, or atom probe tomography. Consequently, specific labels such as η-Ni_3_Ti, η-Ni_3_(Ti, Al), or Ni_3_(Mo, Ti) should be understood as literature-supported working assignments rather than as phases directly confirmed in the present specimens. The SEM/EDS observations were used to distinguish Al/O-rich oxide inclusions from Ti- or Ti/Ni-enriched features and particles, but not to quantify or crystallographically identify nanoscale precipitates. The same limitation applies to the identification of specific phases present in the material’s oxide layer (where, according to EDS, element segregation occurred). The long-term 350°C exposure series was performed for one selected heat-treatment route, and the hybrid-build experiment represents the complete repair/redeposition workflow rather than isolating the effects of process interruption, grinding, reinsertion, and interfacial bonding. Fatigue and thermal-fatigue performance, including matched comparisons with 18Ni-300, remain outside the scope of this study and should be addressed in future work.

## Resource availability

### Lead contact

Further information and requests for resources should be directed to and will be fulfilled by the lead contact, Ludmila Kučerová (skal@fst.zcu.cz).

### Materials availability

This study did not generate new, unique reagents. The M789 AMPO powder used in this work is commercially available from Böhler Edelstahl.

### Data and code availability

• Source data have been deposited at Zenodo and are publicly available at https://doi.org/10.5281/zenodo.18148088.
• This paper does not report original code.
• Additional information required to reanalyze the data reported in this paper is available from the lead contact upon reasonable request.
• A preprint copy is available at https://doi.org/10.5281/zenodo.18144545.

## Acknowledgments

This publication was supported by the project “Mechanical Engineering of Biological and Bio-inspired Systems”, funded as project no. CZ.02.01.01/00/22_008/0004634 by the Programme Johannes Amos Comenius, call Excellent Research.

## Author contributions

L.K.: Conceptualization, funding acquisition, methodology, project administration, supervision, visualization, writing – original draft, writing – review and editing. M.A. **–** methodology, investigation. A.M. **–** investigation, writing – review and editing. P.Ž. – investigation, writing – review and editing. M.K. – investigation, writing – review and editing. J.Š. – investigation, funding acquisition.

## Declaration of interests

The authors declare no competing interests.

## Declaration of generative AI and AI-assisted technologies in the writing process

During the preparation of this work, the authors used ChatGPT (OpenAI) and Grammarly to support language editing and readability improvements. These tools were used only for linguistic correction and stylistic refinement and not to generate scientific content. After using these tools, the authors reviewed and edited the content as needed and take full responsibility for the content of the published article.

## STAR★Methods

### Key resources table

**Table:** 

REAGENT or RESOURCE	SOURCE	IDENTIFIER
**Chemicals, peptides, and recombinant proteins**		

M789 AMPO powder	Böhler Edelstahl	M789 AMPO; composition in Table 1

**Deposited data**		

Source data	Zenodo	https://doi.org/10.5281/zenodo.18148088

**Software and algorithms**		

AZtec EBSD/EDS acquisition software	Oxford Instruments	Acquisition software
Tango EBSD post-processing software	Oxford Instruments	Post-processing software
TOPAS 5	Bruker	XRD/Rietveld refinement software

**Other**		

SLM 280 HL printer	Nikon SLM Solutions AG	LPBF system

### Method details

#### Material and feedstock

Commercial M789 AMPO powder supplied by Böhler Edelstahl was used as received. The supplier-declared feedstock composition and the measured composition of the consolidated LPBF material are reported in Table 1, and powder morphology, particle-size statistics and powder cross-sections are shown in Figure 1. Because the present work focuses on post-processing response, long-term thermal stability and LPBF repairability of consolidated M789 rather than on powder-lot qualification, no additional accredited feedstock analysis was performed.

#### Material analysis

The microstructure was evaluated using an Olympus GX51 light microscope (SM) and scanning electron microscopes (SEM): Tescan VEGA3 and Zeiss EVO MA25. Chemical composition analysis by energy-dispersive spectroscopy (EDS) was performed on a Zeiss Crossbeam 340 SEM, along with electron backscatter diffraction (EBSD). Unless stated otherwise, EBSD was performed using a NordlysNano detector (Oxford Instruments). Data were acquired with AZtec (Oxford Instruments) at an accelerating voltage of 20 kV, a working distance of 11.5 mm, and a specimen tilt of 70°. Phase matching and indexing were conducted using the center detection mode, with 12 bands and a Hough transform resolution of 60. Maps were collected at step sizes of 0.25 μm (1,000×), 0.08 μm (3,000×), 0.05 μm (5,000×) and 0.03 μm (10,000×). EBSD data were post-processed using Tango (Oxford Instruments) employing a 3 × 3 kernel and a misorientation threshold angle of 5°.

The chemical composition of the consolidated LPBF material was measured by optical emission spark spectrometry using a TASMAN Q4 spectrometer. The powder composition was taken from the supplier’s certificate.

Detailed SEM/EDS and EBSD characterisation was concentrated on representative, decision-relevant conditions rather than applied to every point in the heat-treatment matrix. The as-built condition was analyzed as the processing baseline. For the heat-treatment map, the selected states were solution annealing at 1000°C/1 h, solution annealing plus aging at 1000°C/1 h + 500°C/3 h, and direct aging at 500°C/3 h, because these states represent, respectively, homogenisation without precipitation hardening, the manufacturer-recommended two-step route, and a near-peak, practically relevant direct-ageing route. For the thermal-exposure study, detailed cross-sectional oxidation/EDS characterisation was performed at 24 h and 2880 h to compare the early stage of oxide-scale formation with the longest exposure condition. For hybrid samples, EBSD/KAM analysis was performed on the directly aged hybrid condition, comparing the interface with the re-deposited material away from the interface.

The grain size of the as-built M789 steel was evaluated from EBSD maps of transverse-polished cross-sections from samples manufactured in the Z build direction. Grain size was quantified using the linear intercept method. Sets of parallel test lines were superimposed in two mutually perpendicular directions to assess possible directional differences in the printed microstructure. The line spacing was 5 μm in the horizontal direction and 7 μm in the vertical direction. Grains were defined using a critical misorientation angle of 10°. Approximately 990 intercepts were evaluated in the horizontal direction and 730 intercepts in the vertical direction.

The samples were etched in an HF: HNO_3_: glycerine mixture (2: 1: 2) and examined using secondary electrons (SE). For detailed analysis of fine particles and chemically enriched features, polished, unetched surfaces were observed using backscattered electrons (BSE).

X-ray diffraction (XRD) was performed on a Bruker D8 Discover diffractometer using the standard Bragg-Brentano geometry with theta-theta measurements. A copper X-ray tube with a Kα1 wavelength of 1.54056 Å was used as the X-ray source. The measurements were performed identically for all samples over the 30–105° 2θ range, with a step of 0.025°, using UBC collimators combined with 1 mm and 2 mm polycapillaries. The measured diffraction records were subsequently processed in the DiffracEVA program. The evaluation of the retained austenite fractions and lattice parameters was performed using the TOPAS 5 program. The retained-austenite fraction was obtained directly from the refinement, while the α/α′ matrix fraction was reported as the balance to 100%. Nanoscale precipitates and oxide particles were not included as independently quantified XRD phases because their size and/or fraction were below the reliable quantification capability of the applied laboratory XRD setup.

The oxide-layer thickness after thermal exposure was quantified by image analysis of polished cross-sections. Optical micrographs acquired with a 10× objective were used for the evaluation. For each condition, 10 images were analyzed, and three thickness measurements were taken from each, yielding a total of 30 measurements. The values reported in this work correspond to the arithmetic mean of these measurements.

Tensile testing was carried out on a Zwick/Roell Z250 universal testing machine at a strain rate of 0.0067 s^−1^. Two sample geometries were used. Flat herringbone small samples (Figure 2A) with a 5 mm gauge length were employed to evaluate the influence of heat-treatment parameters on mechanical properties and to assess long-term stability at 350°C. In these test series, the large number of samples required justified the use of small samples to reduce material consumption and AM build time. The second geometry comprised larger round samples (Figure 2B) with a 4 mm gauge diameter, machined from samples deposited onto a previously printed M789 part. Here, the larger gauge section ensured that the interface did not occupy the entire (or a substantial) portion of the gauge length, enabling assessment of whether the interface represents the weakest region of the hybrid part. For the small flat samples, five tensile tests were performed for each condition; for the larger round as-built reference samples, six tests were conducted, whereas three tensile tests were performed for each heat-treated hybrid condition, as described in the heat-treated hybrid-condition results. Unless otherwise stated, tables report average values. The two geometries yield systematically different absolute values, with small flat samples showing higher elongation and slightly lower yield strength than the larger round samples for nominally identical conditions; the mechanistic origin of this offset is examined in the mechanical properties section, and comparisons across heat-treatment conditions are therefore kept within a single geometry to preserve internal consistency.

Impact toughness was measured on six samples using the Charpy V-notch test on standard samples (10 × 10 × 55 mm, V-notch depth 2 mm) with a 300 J pendulum. Hardness was determined by Vickers indentation under a 10 kg load (HV10). Microhardness mapping in hybrid samples (across the joint region) was performed using a 100 g load (HV0.1). All tensile and impact samples were machined from printed semi-finished parts (cylinders or prisms), so the as-printed surface was removed before testing.

#### Additive manufacturing

Additive manufacturing was performed on an SLM 280 HL printer (Nikon SLM Solutions AG, Figures 3A–3C). The LPBF parameters were selected from a preliminary internal processability screening, as described in Methods S1. Since the present work focuses on post-processing, thermal stability and repairability rather than on LPBF process-window optimisation, only the final parameter set is reported here: laser power *p* = 350 W, scanning speed v = 811 mm/s, volumetric energy density E = 90 J/mm^3^, layer thickness 40 μm, and hatch spacing 0.12 mm.

The hybrid-build procedure was designed as an application-oriented simulation of LPBF repair/redeposition rather than as a factorial study of process interruption alone. In this configuration, the interface condition necessarily reflects the combined effects of process interruption, removal and reinsertion of the build plate, grinding-induced surface preparation and residual-stress modification, possible surface oxidation or contamination before redeposition, realignment of the platform, and the thermal cycle imposed by the second deposition step. This differs from a simple paused build that remains inside the machine without surface preparation. Therefore, hybrid samples are used here to assess the feasibility and mechanical performance of the complete repair/redeposition route, rather than to isolate the individual contributions of each process step.

In the first step, cylindrical foundations with a height of 45 mm were additively manufactured using the parameters described above. The as-built cylinders after powder removal are shown in Figure 3A. The build plate carrying the printed cylinders was then clamped on a grinder, and the cylinder tops were ground to the required surface quality and flatness. This step ensured a uniform cylinder height and provided top surfaces parallel to the build plate, both of which are essential for the subsequent deposition step. The printed parts were not protected from air exposure during the entire period in which the build plate was outside the build chamber, including handling, surface grinding, and subsequent cleaning. After grinding, standard cleaning and degreasing were performed to remove residual cutting fluid, with rinsing in isopropanol as the final cleaning step. The build plate with the printed cylinders remained outside the build chamber for approximately 6 h before re-deposition. The build plate was then reinserted and aligned in the SLM 280 HL machine, and the remaining 45 mm of the final hybrid part was deposited. As in the insert repair procedure, the first layer was exposed to the laser twice to promote bonding between the new material and the foundation. After fabrication, the samples were cut from the build plate, the support structures were removed, the surfaces were ground, and the samples were tested. The transition between the two deposition stages is clearly visible on the sample surface (see arrow in Figure 3B).

#### Heat treatment and exposure

As recommended by the M789 powder manufacturer, post-processing heat treatment is required to achieve optimum properties after AM. The recommended procedure consists of two steps: solution annealing at 1000°C for 1 h to homogenise the microstructure, followed by aging at 500°C for 3 h. To clarify the influence of heat-treatment parameters on the resulting microstructure and mechanical behavior, a range of annealing and aging temperatures and holding times was investigated.

Three heat-treatment modes were applied: aging only, solution annealing only, and a two-step treatment combining solution annealing with subsequent aging. For solution annealing, three temperatures in the range 900°C–1100°C and holding times of 0.5–1.5 h were examined, with emphasis on the extent of microstructural homogenisation. For aging, temperatures between 400°C and 560°C and holding times of 1–5 h were studied, with particular focus on precipitation hardening and its effects on mechanical properties and microstructure. Finally, selected solution-annealing conditions were combined with aging at 500 or 520°C to evaluate the response to the full two-step heat-treatment route. Heat treatment was applied to samples printed in the Z direction. An overview of the tested heat treatment parameters is provided in Table S1.

One anticipated application of M789 is the production of molds for pressure die casting of non-ferrous alloys such as Zn and Al, where mold temperatures during long-term service can reach approximately 350°C. Accordingly, the thermal stability of additively manufactured M789 was evaluated at this temperature. The long-term exposure was performed for the manufacturer-recommended benchmark condition, 1000°C/1 h + 500°C/3 h, which provides a practically relevant combination of strength, hardness and microstructural homogenisation. This experiment was designed as a stability assessment of the selected conditions under prolonged exposure at 350°C, rather than as a systematic exposure matrix covering multiple heat-treatment routes.

The heat-treated samples were exposed in a furnace at 350°C in air, without a protective atmosphere, for 24 h, 160 h, 720 h, and 2880 h. The reference consisted of heat-treated samples that received no additional thermal exposure. All samples were printed in the Z direction.

### Quantification and statistical analysis

Statistical analyses were performed on replicate mechanical-property datasets to support the interpretation of build-direction effects, heat-treatment response, thermal-exposure effects, and hybrid-build performance. One-way analysis of variance (ANOVA) was used to test the effect of build direction, heat-treatment condition, and thermal-exposure time on the measured mechanical properties. For the heat-treated samples, the heat-treatment regime was treated as a categorical factor, because the investigated schedules did not constitute a complete factorial temperature × time design. Therefore, no two-way ANOVA for temperature × holding time was applied. When comparisons against a reference condition were required, Welch’s pairwise tests were used with Bonferroni correction. Direct comparisons between continuously printed and hybrid/redeposited samples were evaluated using Welch’s two-sample t-tests. Unless otherwise stated, statistical significance was assessed at α = 0.05, and data are reported as mean ± standard deviation.
