# Supplementary material for: From heat-treatment maps to repairable tooling of LPBF-produced M789 maraging stainless steel
Source: iScience. 2026 Sep 1;29(9):117196. doi: 10.1016/j.isci.2026.117196 (PMC13571156; doi:10.1016/j.isci.2026.117196)
Supplement: Supplementary file 1 [file mmc1.pdf]

iScience, Volume ■■

Supplemental information

From heat-treatment maps to repairable tooling

of LPBF-produced M789 maraging stainless steel

Ludmila Kučerová, Michal Ackermann, Andrea Málková, Jiří Šafka, Pavel Žlábek, and Michal Kaufman

Supplemental information

Methods S1. Preliminary LPBF screening identified the processing parameters used for all reported specimens.

The LPBF parameters used in this study were selected through an internal preliminary processability screening of M789 on the SLM 280 HL system. The screening was designed to identify a robust parameter set for producing dense specimens for the subsequent heat-treatment, thermal-stability, and repairability experiments, rather than to define a complete process window. Selection criteria included process stability, metallographic evidence of lack of fusion or excessive porosity, and manufacturability of the required specimen geometries. The final parameter set used for all specimens was laser power $P = 350$ W, scanning speed $v = 811$ mm/s, volumetric energy density $E = 90$ J/mm^3, layer thickness = 40 µm, and hatch spacing = 0.12 mm. The complete screening matrix and defect analysis are outside the scope of this study.

Supplementary Table S1. Heat-treatment conditions determine the mechanical response of LPBF-produced M789.

Source data for Figure 5. Heat-treatment regimes and corresponding mechanical properties of LPBF-produced M789 are reported as mean ± SD. Data were obtained from small flat specimens (n = 5 per condition); TE, total elongation; UTS, ultimate tensile strength; YTS, yield tensile strength.

Heat treatment	UTS [MPa]	σ_{UTS} [MPa]	YTS [MPa]	σ_{YTS} [MPa]	TE [%]	σTE [%]	HV10 [-]	σ_{HV10} [-]
400 °C/3 h	1258	7	1108	7	21	0.5	426	2
440 °C/3 h	1560	11	1429	9	9	5	525	2
480 °C/3 h	1694	10	1564	26	11.6	4.2	557	8
500 °C/3 h	1767	9	1655	11	11.8	1.2	562	4
520 °C/1 h	1733	19	1623	46	13.3	0.6	547	3
520 °C/2 h	1744	2	1669	24	12.7	0.8	551	2
520 °C/3 h	1797	7	1702	8	13.4	0.3	572	3
520 °C/5 h	1773	8	1651	46	13.3	1.2	556	4
540 °C/3 h	1747	1	1659	10	13.5	1.1	553	2
560 °C/3 h	1472	11	1396	12	19.0	1	487	2
900 °C/1 h	978	7	710	23	23.0	0.6	318	4
1000 °C/0.5 h	958	5	723	23	22.3	0.6	301	7
1000 °C/1 h	957	4	717	7	21.6	0.7	313	3
1000 °C/1.5 h	958	2	711	39	22.7	0.5	310	2
1100 °C/1 h	953	4	702	21	22.6	0.3	307	4
900 °C/1 h + 520 °C/3 h	1720	6	1647	10	13.5	0.6	533	6
1000 °C/0.5 h + 520 °C/3 h	1722	5	1634	20	14.3	0.6	549	16
1000 °C/1 h + 520 °C/3 h	1719	2	1628	11	13.5	0.2	553	8
1000 °C/1.5 h + 520 °C/3 h	1666	5	1546	13	15.8	1	517	7
900 °C/1 h + 500 °C/3 h	1662	36	1521	64	12.8	0.9	528	12
1000 °C/0.5 h + 500 °C/3 h	1745	11	1646	4	10.8	6.2	548	5
1000 °C/1 h + 500 °C/3 h	1735	5	1610	23	12.1	0.6	558	7
1000 °C/1.5 h + 500 °C/3 h	1690	10	1556	20	13.2	1.5	549	4
1000 °C/1 h + 500 °C/2 h	1710	6	1594	10	7.5	4.2	552	11

Supplementary Table S2. Heat treatment changes the retained-austenite fraction in LPBF-produced M789.

Retained-austenite fractions were determined by XRD/Rietveld refinement; the α/α′ matrix fraction was calculated as the balance to 100%. Nanoscale ageing precipitates and Ti/Al-rich oxide particles were below the reliable detection/quantification limit of the laboratory XRD setup and were therefore discussed using SEM/EDS evidence, mechanical response, and literature-supported interpretation rather than XRD phase quantification.

Condition	retained austenite [%]	α/α′ matrix [%]
900 °C / 1 h	<2	>98
1000 °C / 0.5 h	n.d. / below detection	≈100
1000 °C / 1 h	n.d. / below detection	≈100
1000 °C / 1.5 h	n.d. / below detection	≈100
500 °C / 3 h	5.8	94.2
540 °C / 3h	5.8	94.2
1000 °C / 0.5 h + 500 °C / 3 h	5.1	94.9
1000 °C / 1 h + 500 °C / 3 h	5.6	94.4
1000 °C / 1.5 h + 500 °C / 3 h	6.6	93.4
As-built	3.2	96.8
